# Phosphate and Carbonate in the Biomineralization of Chicken Eggshells and the Increase in Eggshell Thickness Through Nanodroplet Addition

**DOI:** 10.3390/ani16193023

**Published:** 2026-09-25

**Authors:** Antonio Valadão Cardoso, Rodrigo Novaes Ferreira, Maria Sylvia Dantas, Leonardo H. R. Dos Santos, Ana Paula Gomes, Lara Luz de Assis

**Affiliations:** 1 School of Design, State University of Minas Gerais (UEMG), Belo Horizonte 30140-091, MG, Brazil; 2 Microscopy Center, Federal University of Minas Gerais (UFMG), Belo Horizonte 31270-901, MG, Brazil; kinects@yahoo.com.br; 3 Metallurgy Department, School of Engineering, Federal University of Minas Gerais (UFMG), Belo Horizonte 31270-901, MG, Brazil; dantas.sylvia@gmail.com; 4 Department of Chemistry, Federal University of Minas Gerais (UFMG), Belo Horizonte 31270-901, MG, Brazil; leonardohrs@qui.mest.ufmg.br (L.H.R.D.S.); lluz2024@ufmg.br (L.L.d.A.); 5 Laboratory of Characterization and Processing of Nanomaterials, Federal University of Minas Gerais (UFMG), Belo Horizonte 31270-901, MG, Brazil; anasbisgouslly@gmail.com

**Keywords:** eggshell, biomineralization, calcium phosphate, calcium carbonate, *Gallus gallus domesticus*

## Abstract

The presence of hydroxyapatite (HAp) in the Cuticle of laying-hen eggshells was investigated through a comprehensive characterization of its morphology, composition, crystallographic structure, and thermal behavior. Comparison with bone-derived hydroxyapatite confirmed the similarity of the mineral phase. Examination of the same region at different imaging contrasts revealed unidirectional (nanofibrous) calcite growth within the Vertical Layer (VL) and Palisade Layer (PL). Shell thickening in these layers appears to occur through an additive mechanism involving the successive deposition of nanodroplets containing, according to our hypothesis, the mineral phase, water, and organic components. This multiphasic system generates lamellae that progressively increase in thickness through the continuous incorporation of new nanodroplets onto the pre-existing surface. Such a nanodroplet-mediated growth mechanism also provides a plausible explanation for the formation of the micropores observed in the VL and PL. The proposed additive mechanism is further supported by the presence of nanohemispheres attached to growing lamellae and the similar diameters of cuticle nanospheres and Vertical Layer nanohemispheres. Fractures observed in the Vertical Layer indicate structural continuity between the Cuticle and the VL, suggesting that additive growth involves a continuous supply of HAp, possibly across the entire uterine surface, followed by a previously undescribed process in which calcium phosphate nanospheres dissolve and/or transform into calcium carbonate nanofibers.

## Simple Summary

Inside the uterus of the laying hen, in less than a day, microscopic particles of calcium-containing minerals, under the action of substances present in the uterine fluid, dissolve and, as nanodroplets, precipitate onto the inner membrane of the egg (the one we see when we crack an egg). Thus, they nucleate a solid shell that will protect the egg white and yolk and, later, a developing embryo. Imagine the entire surface of the hen’s uterus continuously expelling these vesicles, which react and combine with substances in the uterine fluid to form nanodroplets containing calcium mineral. These nanodroplets precipitate continuously until the egg reaches sufficient thickness to form a very porous but also very strong solid. As our study showed, mineral fibers arise from the nanodroplets and embedded in the organic matrix and form a composite. This construction of a solid within the uterine fluid differs from any engineering known or developed by us. By combining mineral and organic components and using sophisticated nanodroplets, bird eggs show that evolution has learned to use basic phenomena occurring in calcium minerals to build eggs and shells, as well as skeletons for complex beings such as humans.

## 1. Introduction

Laying hens accomplish the remarkable feat of biomineralizing an eggshell within a period of 18–20 h, producing a shell with a thickness ranging from 0.3 to 0.4 mm [1,2,3,4]. This remarkable capacity occurs throughout the laying period of the hen and may last for two to three years, depending on the bird’s living conditions [5]. The production of approximately 500 eggs during the productive lifetime of commercial laying hens is currently one of the main targets of genetic breeding programs [6].

Over the past three decades, research on the biomineralization of the eggshell of laying hens (*Gallus gallus domesticus*) has advanced considerably, and the incorporation of concepts and analytical techniques from Materials Science has proved highly fruitful in expanding our understanding of eggshell formation.

In approaching the eggshell as a material, we identify four main areas of investigation: (1) nucleation and growth of calcite from the membrane surrounding the egg white; (2) the organic ultrastructure upon which the mineralized layer develops; (3) shell porosity, including both closed and open pores; and (4) the eggshell cuticle.

It is generally accepted that biomineralization in living systems [7] proceeds through nanometric entities that give rise to hierarchical structures exhibiting interactions and feedback across multiple length scales. This concept has also been proposed for the avian eggshell and differs substantially from the principles governing composite materials [8,9]. It was already observed several decades ago that both the inorganic component (calcite) can nucleate and grow on an organic substrate and, conversely, that macromolecules can adsorb onto a calcitic substrate [10]. This behavior differs fundamentally from conventional composite technology, in which at least two distinct constituents are present: a dispersed phase (particle or fiber) and a continuous matrix. In composite materials, wetting of the reinforcement by the matrix is an essential requirement. In contrast, in the eggshells of birds, chelonians, reptiles, and other vertebrates, proteins appear to influence the thermodynamic phase [11,12,13], the size and morphology of the calcite polycrystals [14], and, in particular, the porosity of the inorganic matrix.

The classification of the eggshell as a composite material appears in many studies describing biomineralization through the heterogeneous nucleation of amorphous calcium carbonate (ACC) on the organic ultrastructure, followed by its transformation into calcite. According to this model, ACC reaches the biomineralization sites (the shell membrane and the palisade layer) within extracellular vesicles [15], whose surfaces are associated with different proteins [16]. As shell thickness increases, successive calcite layers nucleated from the inner eggshell membrane develop into structures known as mammillae, above which the palisade layer is formed. In scanning electron microscopy (SEM) images, a calcite crown can be observed at the apex of each mammilla [17] (see Figure S7 in the Supplementary Materials). Although proteins account for less than 3% of the shell mass, their ultrastructure plays an essential role in calcite nucleation and growth [14]. However, whether the organic and inorganic phases are assembled sequentially or simultaneously remains unclear.

Calcium and phosphorus are supplied primarily through the hen’s diet. An additional source of calcium for eggshell formation is the dissolution of medullary bone [18,19,20], followed by its transport through the bloodstream to the uterus, where biomineralization takes place.

The dissolution of carbonated hydroxyapatite (CHAp) [21] from medullary bone and its transport through the circulatory system remain poorly understood. Several published studies have reported spherical hydroxyapatite nanoparticles on the inner surface of the eggshell cuticle [17,22]. Infrared spectroscopy studies [23] suggest that these nanospheres may be predominantly amorphous. In contrast, the pioneering work of Dennis et al. [17] presented electron microscopy images of hydroxyapatite nanoparticles together with X-ray diffraction evidence confirming their crystalline structure.

More recent studies have emphasized that the nucleation of amorphous and/or crystalline calcium carbonate begins on the inner eggshell membrane, which encloses the albumen and yolk [15,24,25,26]. Investigations specifically focused on the cuticle have mainly addressed the effects of hen age, genetic lineage, and industrial egg-cleaning procedures on its structure and function [23,27].

There is general agreement regarding the presence of phosphate in the cuticle, as reported since the earliest investigations of its composition [28]. At one stage, high phosphate concentrations were proposed to be associated with defects in the biomineralization process and even with premature oviposition [29,30].

Calcium phosphate nanospheres may actually correspond to extracellular vesicles containing a liquid phase enriched with aggregates of amorphous hydroxyapatite, similarly to models proposed for bone biomineralization [31,32].

Some recent studies [33] have followed the hypothesis originally proposed by Dennis et al. [17], suggesting that hydroxyapatite nanospheres present in the cuticle participate in the final stages of calcite biomineralization. Support for this hypothesis is indirect and is mainly based on observations indicating that phosphate inhibits calcite nucleation [34].

However, more recent investigations [35] indicate that phosphate does not necessarily inhibit calcium carbonate crystallization.

In natural environments, phosphate is known to promote heterogeneous nucleation and crystallization of calcite [36].

Studies of eggshell microstructure and nanostructure have also demonstrated that shell porosity exhibits a hierarchical organization. Zhou et al. [37] described a large population of pores distributed throughout the shell, exhibiting bubble-like morphologies and relatively uniform submicrometric diameters. Eggshell porosity has attracted considerable interest from both industrial and scientific perspectives [38,39,40]. Using micro-computed tomography (micro-CT), Riley et al. [41] demonstrated that voids occupy approximately one-third of the total shell volume. These voids are distributed throughout the shell and clearly illustrate the gas-conducting function of open pores, which traverse the entire shell and originate from the spaces formed between adjacent mammillae. A locally thickened region of the cuticle, known as the pore plug, acts as a cap sealing these pores. Atomic Force Microscopy (AFM) has also been successfully employed to characterize eggshell porosity [42].

Despite the substantial advances achieved over the past decades, several crucial aspects of eggshell biomineralization remain unexplored or insufficiently understood. One of these unresolved questions concerns the chemical nature of the phosphate compound present on the inner side of the eggshell cuticle. Even more importantly, the interaction between the micrometric calcium phosphate layer and the underlying calcite (CaCO_3_) layer has never been directly investigated. Does calcite grow through the dissolution of calcium phosphate followed by the precipitation of calcium carbonate?

In the present study, we provide direct visualization of these phenomena through a detailed ultrastructural investigation using scanning electron microscopy (SEM) combined with energy-dispersive X-ray spectroscopy (EDS). Backscattered electron (BSE) imaging, which provides greater compositional contrast than secondary electron (SE) imaging, enabled the analysis of morphological features relevant to the eggshell biomineralization process, particularly by distinguishing predominantly inorganic from organic regions. To investigate the transition between hydroxyapatite present in the cuticle and calcite in the Vertical Layer (VL), micro-Raman and micro-FTIR techniques proved particularly important, allowing the spatial distribution of these two mineral phases to be distinguished. The combination of these techniques with thermogravimetric analysis (TG) and X-ray diffraction (XRD), whose complementary results are presented in the Supplementary Materials, enabled eggshell characterization at different scales and from complementary morphological, compositional, and structural perspectives. This complementarity, together with the results obtained, supports the multitechnique approach adopted in this study.

We hypothesized that hydroxyapatite is involved in chicken eggshell formation and is associated with the initial stages of calcite mineralization. To test this hypothesis, the main objective of this study was to investigate the structural and compositional organization of the different eggshell layers, with particular emphasis on the cuticle and its relationship with calcite mineralization. For this purpose, we used a multimodal approach combining SEM/BSE/EDS, Raman spectroscopy, FTIR, XRD, and TGA. Specifically, we addressed three research questions: (i) What are the morphology and composition of the cuticle and its interface with the Vertical Layer (VL)? (ii) How does calcite growth evolve from one-dimensional (1D, fibers) to two-dimensional (2D, plates) and three-dimensional (3D, volume) structures? and (iii) Does eggshell thickening involve a sequential process of composite formation, in which calcite fibers form first and the organic matrix is subsequently deposited around them?

## 2. Materials and Methods

### 2.1. Specimen Collection and Preparation

#### 2.1.1. Eggshells

Egg samples were obtained by purchase from local supermarkets (10 white-shelled eggs and 10 brown-shelled eggs, both marketed as Extra size) of the commercial brand Mantiqueira (Itanhandu, Minas Gerais, Brazil). According to information provided by the producer (https://mantiqueirabrasil.com.br/), the commercial laying hens belong to different genetic strains, including Hy-Line and Lohmann, with laying periods ranging from 18 to 90 weeks of age. The eggs had an average weight of (63.3 ± 0.1) g, whereas the cleaned and dried eggshells presented an average weight of (7.2 ± 0.1) g. The average eggshell thickness of the brown eggs, measured by SEM, was (357 ± 7) μm and is presented in Table 1 (Section 3). Thus, a total of 20 eggshells from 20 individual eggs were used throughout the study.

Immediately after breaking the eggs and removing their contents, the shells were washed under running tap water for several minutes, followed by careful visual inspection to ensure that no albumen, yolk, or membrane residues remained adhered to either the inner or outer shell surfaces as a result of the breaking procedure. Subsequently, the shells were dried in an electric oven (Brastemp, São Paulo, Brazil) at 60 °C for 3 h.

After drying, the shells were stored in plastic bags and kept for SEM-EDS, Raman spectroscopy, FTIR, and XRD analyses. No additional thermal or chemical treatment was performed on these samples at this stage, which constituted the main part of the study.

For a second stage of the study, eggshells from five different eggs were calcined in air at 150, 350, 550, 750, and 950 °C using an electric furnace (Oga, Belo Horizonte, Brazil). After thermal treatment and cooling to room temperature, part of the material was ground in a porcelain mortar (Chiarotti, São Paulo, Brazil), whereas the remaining material was kept as shell fragments, which became highly fragile after calcination. The powdered samples and shell fragments were subsequently transferred to 15 mL Falcon tubes with screw caps and stored for subsequent thermogravimetric (TG), FTIR, and XRD analyses.

#### 2.1.2. Micro-Raman and Micro-FTIR Analyses

For micro-Raman and micro-FTIR analyses, eight eggshell fragments were prepared, four for each technique. The fragments were embedded in acrylic resin, ground, and polished to expose the entire shell thickness for analysis under the optical microscope coupled to each instrument.

A second preparation method consisted of embedding only part of the eggshell fragment, followed by very gentle grinding and polishing and subsequent cleaning using extra-soft bristle brushes.

#### 2.1.3. Chicken Bones

Chicken thighs were purchased from local supermarkets, and the femur bones were completely separated from the surrounding soft tissues. The bones were cleaned using a plastic-bristle brush under running tap water for several minutes to remove any adhering biological material. They were subsequently dried in an electric oven (Brastemp, São Paulo, Brazil) at 60 °C for 3 h.

After drying, the bone samples were immersed in a 2.5 wt.% sodium hypochlorite solution for 72 h. The samples were then rinsed, dried again at 60 °C for 3 h, fractured into small pieces, transferred to Falcon tubes, and stored under refrigeration until Raman and FTIR spectroscopy analyses.

#### 2.1.4. Bovine Hydroxyapatite

Bovine hydroxyapatite was prepared in the laboratory from bovine tibiae supplied by a local butcher and previously cut into cylindrical sections approximately 3–4 cm in length. Initially, the bone pieces were cleaned with a brush under running tap water and subsequently heated in an electric oven (Brastemp, São Paulo, Brazil) at 60 °C for 6 h to facilitate the removal of residual organic material from the medullary cavity. The samples were then brushed again under running water and dried for an additional 3 h at 60 °C.

The dried bone samples were immersed in a 2.5 wt.% sodium hypochlorite solution for 96 h, followed by drying at 60 °C for 6 h. The treated bones were fractured into small fragments and stored in Falcon tubes under refrigeration. For X-ray diffraction analysis (Supplementary Materials, Figure S8), part of the material was ground into a fine powder.

### 2.2. X-Ray Diffraction (XRD)

The crystalline phases present in the eggshell samples were identified by X-ray diffraction (XRD) using a multipurpose diffractometer (Anton Paar, XRDDynamic 500, Graz, Austria) equipped with a Cu Kα radiation source. The operating conditions were 40 kV, 50 mA, and a 2θ scanning range from 10° to 90°.

Phase identification was performed using the Inorganic Crystal Structure Database (ICSD) accessed through the Cambridge Crystallographic Data Centre (CCDC) software platform, Web version 5.5.0, Data Release 2025.2 (FIZ Karlsruhe, Germany).

Before analysis, the eggshells were carefully washed once again with deionized water using a soft brush and allowed to air-dry. XRD analyses were performed on finely powdered eggshell samples prepared by grinding the shells in a porcelain mortar and pestle, as described in Section 2.1.

### 2.3. Scanning Electron Microscopy (SEM) and Energy-Dispersive X-Ray Spectroscopy (EDS)

Eggshell samples were prepared and analyzed at the Microscopy Center of the Federal University of Minas Gerais (CM-UFMG, Belo Horizonte, Brazil) using three scanning electron microscopes:

(a) FEI Quanta 3D FEG DualBeam (FEI Company, Hillsboro, OR, USA), equipped with backscattered electron (BSE) and energy-dispersive X-ray spectroscopy (EDS) detectors;
(b) Thermo Fisher APREO-2C (Thermo Fisher, Waltham, MA, USA), equipped with BSE and EDS detectors;
(c) FEI Quanta 200 FEG (FEI Company, Hillsboro, OR, USA).

Three eggshell specimens obtained from three different eggs were used for SEM investigation. After cleaning and drying, the samples were coated with a 50 nm graphite layer to allow high-quality EDS analysis. A fourth specimen, prepared under the same conditions, was coated with a gold–palladium alloy to optimize secondary electron imaging. Before and after microscopy sessions, all specimens were stored in a low-humidity desiccator.

A total of six microscopy sessions, each lasting approximately 2 h, were carried out to investigate the eggshell thickness and its ultrastructure. Elemental maps of carbon (C), oxygen (O), calcium (Ca), phosphorus (P), magnesium (Mg), and sulfur (S) may exhibit slight differences in color among figures because the SEM-EDS analyses were performed during different microscopy sessions.

### 2.4. Raman Spectroscopy

Raman spectroscopy analyses were performed using two instruments.

#### 2.4.1. Horiba LabRAM HR800

A LabRAM HR800 Raman spectrometer (Horiba Jobin Yvon, Oberursel, Germany), equipped with a 632.8 nm He–Ne laser and an Olympus BX41 optical microscope fitted with 10×, 50×, 100×, and 100× long working distance (LWD) objectives, was used. The laser beam was focused onto an area of approximately 1–2 μm^2^ using the 100× objective. The scattered light was dispersed by a monochromator and detected using a liquid nitrogen-cooled CCD detector.

Spectra were recorded over the 100–4200 cm^−1^ spectral range with a spectral interval of 1.1 cm^−1^. Most measurements were acquired between 80 and 1250 cm^−1^, using an acquisition time of 30 s and 10 accumulations to improve the signal-to-noise ratio.

#### 2.4.2. WITec Alpha300 RA

Additional Raman measurements were performed using a WITec Alpha300 RA Raman spectrometer (Witec, Ulm, Germany) equipped with a 532 nm diode laser, an optical microscope, and an EMCCD detector. The laser beam was focused onto an area of approximately 1 μm^2^.

Spectra were acquired mainly within the 80–1250 cm^−1^ range using a spectral resolution of 1 cm^−1^, a 1800 lines mm^−1^ diffraction grating, a 100× objective, 10 accumulations, and an integration time of 60 s per accumulation.

For both Raman systems, the laser power was carefully adjusted to avoid local heating and possible thermal damage to the samples.

### 2.5. FTIR and Micro-FTIR-ATR Spectroscopy

FTIR and micro-FTIR-ATR analyses were performed on eggshell samples prepared as described in Section 2.1, either as finely powdered material or as shell fragments approximately 2 cm in length. Untreated eggshells and shells thermally treated at 150, 350, 550, 750, and 950 °C were analyzed. Treated chicken femur samples and laboratory-prepared bovine hydroxyapatite were also characterized.

Infrared spectroscopy analyses were carried out using two instruments:

#### 2.5.1. FTIR

An IRSpirit FTIR spectrometer (Shimadzu, Kyoto, Japan) operating in transmittance mode over the 400–4000 cm^−1^ spectral range.

#### 2.5.2. Micro-FTIR-ATR

A Tracer-100 FTIR-ATR spectrometer (Shimadzu, Kyoto, Japan) coupled to an AIM-9000 infrared microscope (Shimadzu, Kyoto, Japan) equipped with both reflectance and attenuated total reflectance (ATR) modules. Measurements were performed over the 700–4000 cm^−1^ spectral range in transmittance mode.

All spectra were acquired at room temperature. Powder FTIR spectra were recorded with a spectral resolution of 2 cm^−1^, whereas micro-FTIR-ATR spectra were obtained with a resolution of 1 cm^−1^. Spectral interpretation was performed using the Cambridge Crystallographic Data Centre (CCDC) database and software platform.

### 2.6. Thermogravimetric Analysis (TGA)

Thermogravimetric analysis (TGA) was performed using a Shimadzu DTG-60/DTG-60H TG/DTA analyzer (Shimadzu, Kyoto, Japan). Approximately 10mg of each sample was placed in an alumina (Al_2_O_3_) crucible and analyzed under a nitrogen atmosphere with a flow rate of 100 mL min^−1^. The heating rate was 10 °C min^−1^, and the temperature range investigated extended from room temperature to 600 °C. The thermogravimetric results are presented in the Supplementary Materials.

### 2.7. Image Analysis (Feret Diameter) and Statistical Analysis

Morphometric measurements of eggshell microstructures were performed on SEM images using ImageJ2 (version 2.16.0/1.54p). Statistical analyses were carried out using StatPlus (version 8.0.4.0).

Differences among the measured structural dimensions were evaluated using the Kruskal–Wallis non-parametric test, followed by Dunn’s multiple-comparison post hoc test with Bonferroni correction. Statistical significance was considered at *p* < 0.05.

The measurements presented in Table 1 were obtained from different SEM images of a single eggshell specimen (identified as sample V2500395). The n value (number of measurements per layer) varied among layers depending on the number of SEM images available for analysis. All measurements were performed using the measurement tools available in the ImageJ2 software.

## 3. Results

### 3.1. SEM–EDS

Figure 1, Figure 2, Figure 3, Figure 4, Figure 5, Figure 6, Figure 7, Figure 8 and Figure 9 present the results obtained by scanning electron microscopy (SEM) and energy-dispersive X-ray spectroscopy (EDS). To provide complementary structural information, images of the same regions were intentionally acquired using two different electron detectors: the secondary electron (SE) detector, which provides detailed topographical information, and the backscattered electron (BSE) detector, which generates compositional contrast based on differences in the average atomic number of the constituent phases. Consequently, surfaces that appear relatively homogeneous in SE images reveal considerably more detailed surface and subsurface features when examined using the BSE detector.

Figure 1 presents SEM images of the entire thickness of the laying-hen eggshell, extending from the cuticle to the inner eggshell membrane. Morphometric measurements (n) were performed on SEM images from a single eggshell specimen using the ImageJ software. The total shell thickness was (353 ± 7) μm (Table 1) and comprised the following regions: (a) cuticle (C), (b) Vertical Layer (VL), (c) Palisade Layer (PL), (d) Mammillary Region (MA), and (e) inner eggshell membrane (M).

Large pore channels traversing the entire shell thickness are visible in the central region of the image. These channels exhibit substantial diameters (approximately 70 μm) both near the inner membrane and close to the cuticle. Their three-dimensional morphology resembles two cones whose apices meet approximately at the middle of the shell thickness.

The mammillae, where calcite (CaCO_3_) nucleation and crystal growth take place, are clearly visible in both images of Figure 1. In Figure 1b, it can be observed that the bases of the mammillae [43] originate from distinct and individualized nucleation sites attached to the outer surface of the inner eggshell membrane. According to the literature, the inner eggshell membrane [44] is actually composed of two collagen-rich membranes, referred to as the inner and outer shell membranes [45,46].

Elemental maps of carbon (C), calcium (Ca), phosphorus (P), oxygen (O), and sulfur (S) in the eggshell, obtained by energy-dispersive spectroscopy (EDS), are shown in Figure 1c. A white arrow indicates the location of phosphorus (P) in the lower part of the cuticle. In the channels that cross the shell, the thickness of the phosphorus-containing layer, also called the pore plug (1), extends further into the shell interior. Carbon (C) appears marked on the upper face of the cuticle and on the inner membrane, whereas calcium (Ca) and oxygen appear marked throughout the shell thickness. Also note the presence of sulfur (S)—due to sulfated polysaccharides [46]—in the inner eggshell membrane.

**Figure 1:**
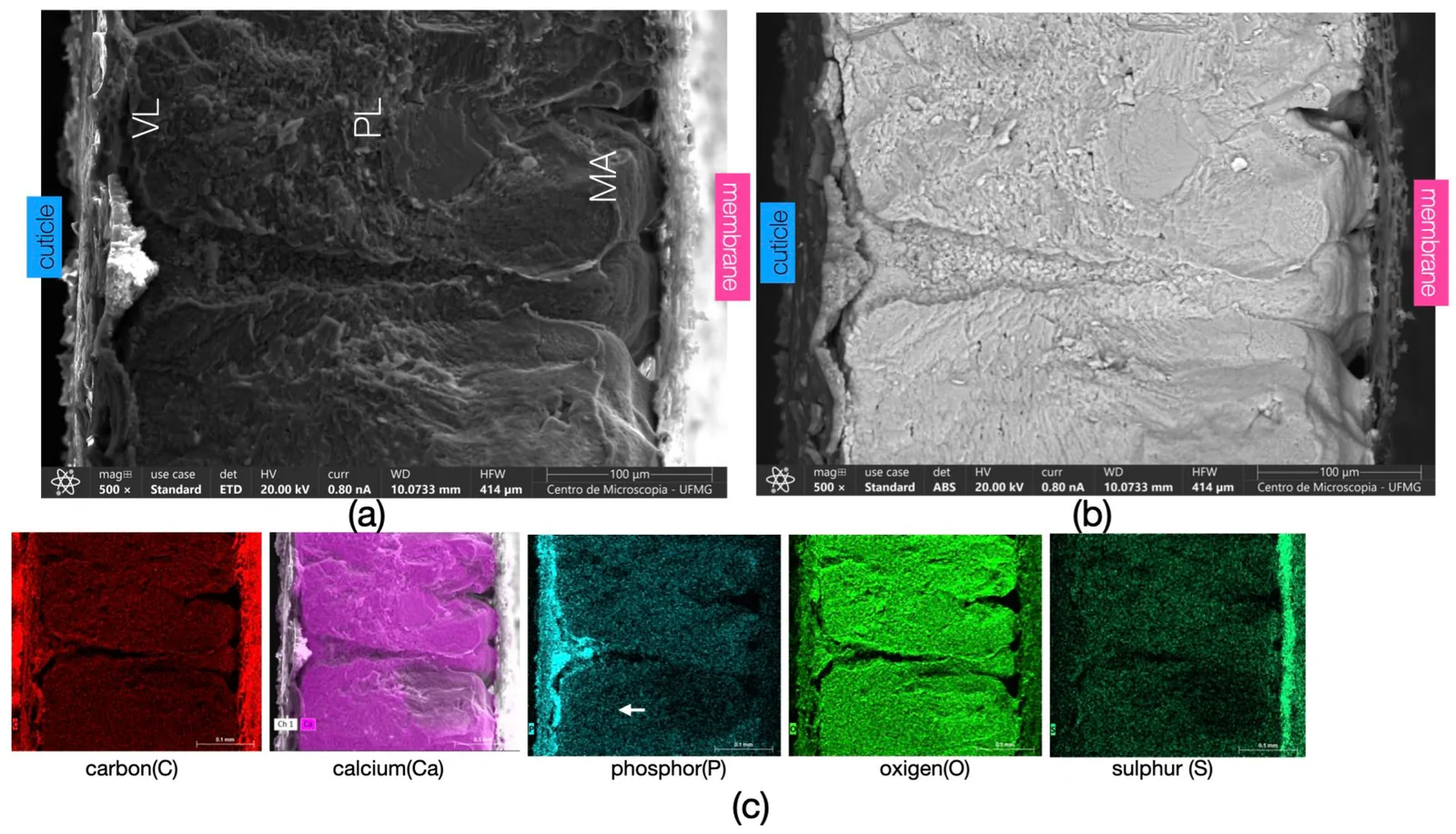
(**a**) Image showing the thickness [(353 ± 7) μm] of a laying hen eggshell (*Gallus gallus domesticus*), from the cuticle (left side) to the membrane (right side), where biomineralization begins. The SE (secondary electron) detector highlights the shell topography. In the central part of the image, voids forming channels/pores can be observed extending uninterruptedly from the cuticle to the membrane. The sequence of the Vertical Layer (VL), Palisade Layer (PL), and Mammillary Layer (MA) is indicated in the panoramic image of the shell (**a**). (**b**) The same region of the same sample imaged using the BSE (backscattered electron) detector, with lighter regions corresponding to elements of higher atomic number. It is possible to observe (left side of the image) that the tips of the mammillae begin at different points attached to the membrane surface. (**c**) Profiles of the most abundant chemical elements in the eggshell: C (carbon), Ca (calcium), P (phosphorus), O (oxygen), and S (sulfur). While carbon (C) and oxygen (O) are found on both faces of the shell (upper part of the cuticle and inner membrane), phosphorus (P) is concentrated in the cuticle and calcium (Ca) throughout the entire biomineralized region. The white arrow in the phosphorus (P) profile indicates a higher concentration of this element on the lower face of the cuticle. Note, in the same image, that the phosphorus-containing layer (pore plug) presents a larger area in the region of the channel/pore that crosses the shell.

Table 2 presents the concentration data (EDS, semi-quantitative) for carbon (C), oxygen (O), magnesium (Mg), phosphorus (P), sulfur (S), and calcium (Ca). The data are divided by the investigated areas (cuticle, cuticle + vertical layer VL, etc.; see the legend on the right side of Table 2). In Table 2, some of the different layers that constitute the eggshell are presented together because, under the experimental conditions used for EDS spectrum acquisition, it was not possible to unequivocally distinguish the contribution of each individual layer.

In Table 2, the two types of sample coating (carbon or gold/palladium) are separated. Carbon coating is the preferred option for EDS because it does not generate interfering peaks, allowing more accurate quantification. On the other hand, gold peaks overlap with those of other elements such as phosphorus. In EDS measurements performed with gold coating (lower part of Table 1), phosphorus is practically absent because the gold peak overlaps it. Figure S6 (Supplementary Materials) shows the overlap of the gold peak with phosphorus in the EDS analysis of the cuticle. This may be one source of the non-detection of phosphorus in vesicles that would transport material for biomineralization [47]. In Table 2, some of the analyzed areas are represented by only one observation or a single pair of measurements. This limitation results from the acquisition conditions during the EDS analysis sessions, which did not allow a larger number of independent measurements to be obtained for all the investigated regions. Consequently, these results should be interpreted as semi-quantitative data representative of the analyzed regions rather than as a statistical assessment of the compositional variability of each layer.

The SEM images in Figure 2 correspond to the cuticle (black arrow in Figure 2(a1)) and to the calcite region immediately below the cuticle (marked by magenta ellipses in Figure 2(a1,b1)). The cuticle thickness was evaluated as (1.9 ± 0.4) μm. Images from left to right correspond to progressively higher magnifications of the same region, focusing on the cuticle (magenta arrows). Cuticle thickness is shown in Figure 2(a2,b3) with black arrows and in Figure 2(a3) with white arrows. Figure 2(a1–a3) was acquired with the SE sensor (topography), whereas Figure 2(b1–b3) was acquired with the BSE sensor, which allows compositional differences to be distinguished as darker or lighter regions depending on the atomic weight of the elements present. Thus, the calcite layer appears light gray, whereas the cuticle appears dark gray. In Figure 2(b1,b2), and especially Figure 2(b3), spheres of different diameters are observed throughout the cuticle.

A magenta asterisk was placed in Figure 2(a3,b3) to show that a fracture in the cuticle region kept the cuticle and the vertical calcite layer (VL) united and intact, while the fracture propagated through the VL. In Figure 2(b3), acquired with the BSE sensor, unidimensional (fibrillar) calcite growth is observed, intimately connected with the base of the cuticle. Additional images showing the continuity between the cuticle and the VL are presented in Figure S8 in the Supplementary Materials.

In Figure 3, the images show the cuticle in the region of the channels presented in Figure 1. These channels occur as a consequence of the mammillae shape: they are wider both in the mammillary region and in the upper part, just below the cuticle. Figure 3(a1) (SE sensor, topography) and Figure 3(a3) (BSE sensor, greater depth in structural detailing), respectively, show that the cuticle has a much greater thickness at this location, forming a “cluster” of spheres. The EDS composition maps shown in Figure 3(a2,b2) confirm phosphorus (P) as one of the elements present in the spheres. Figure 3(b1) (SE sensor) and Figure 3(b3) (BSE sensor), respectively, show in detail the spheres, with similar diameter, that form this “cluster”.

**Figure 2:**
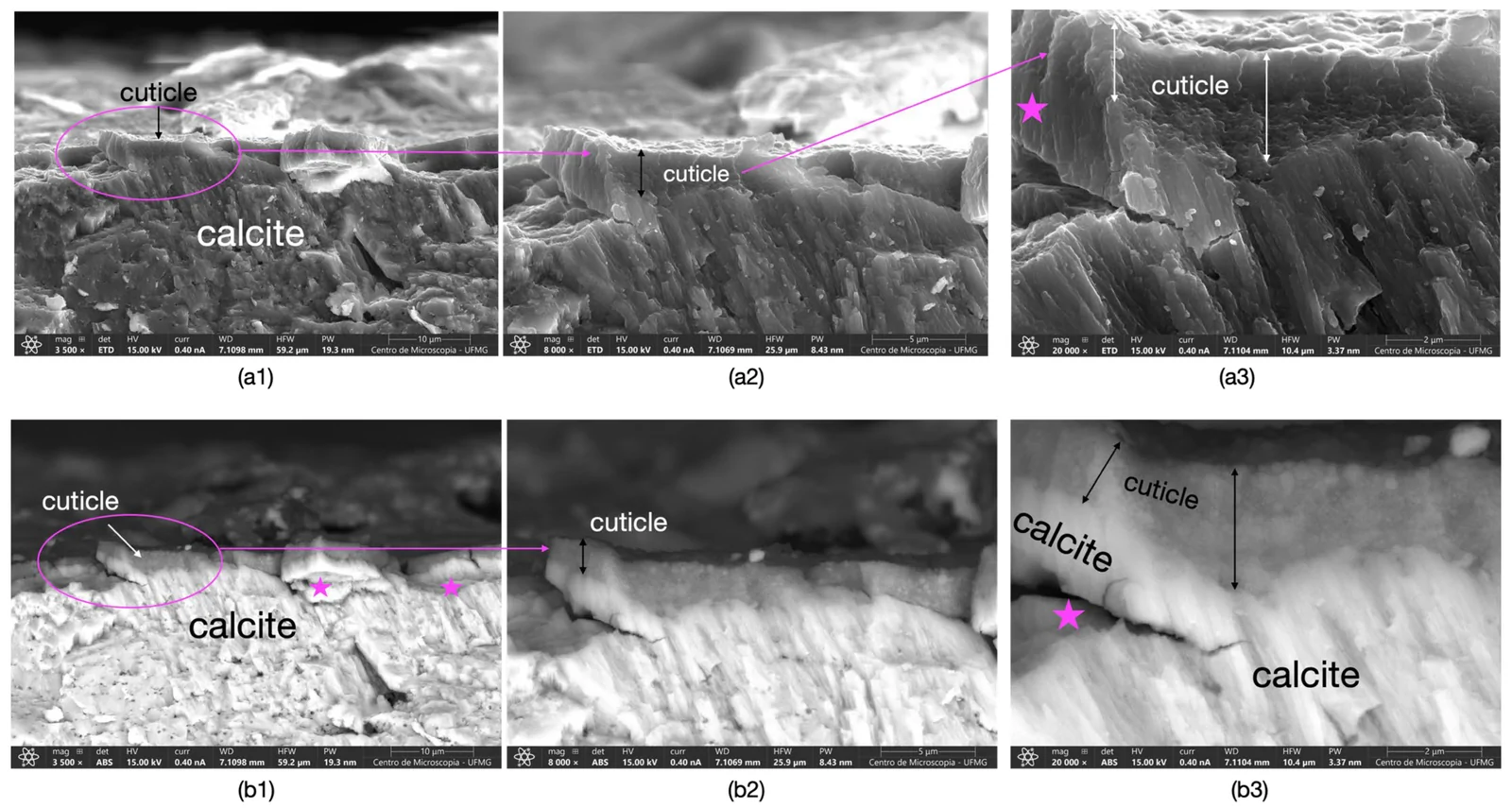
SEM images, at different magnifications, of the same area of the eggshell cuticle: SE detector images (**a1**–**a3**) providing the topography of the eggshell cuticle, and images (**b1**–**b3**) acquired with the BSE detector, providing more detailed images (compositional differences) of the same region. The presence of spheres in the cuticle is visible especially in images (**b2**,**b3**). Spherical shapes in the cuticle region can also be observed in image (**a3**). A magenta star was placed at locations where fractures evidence continuity between the cuticle and the underlying calcite vertical layer (VL).

**Figure 3:**
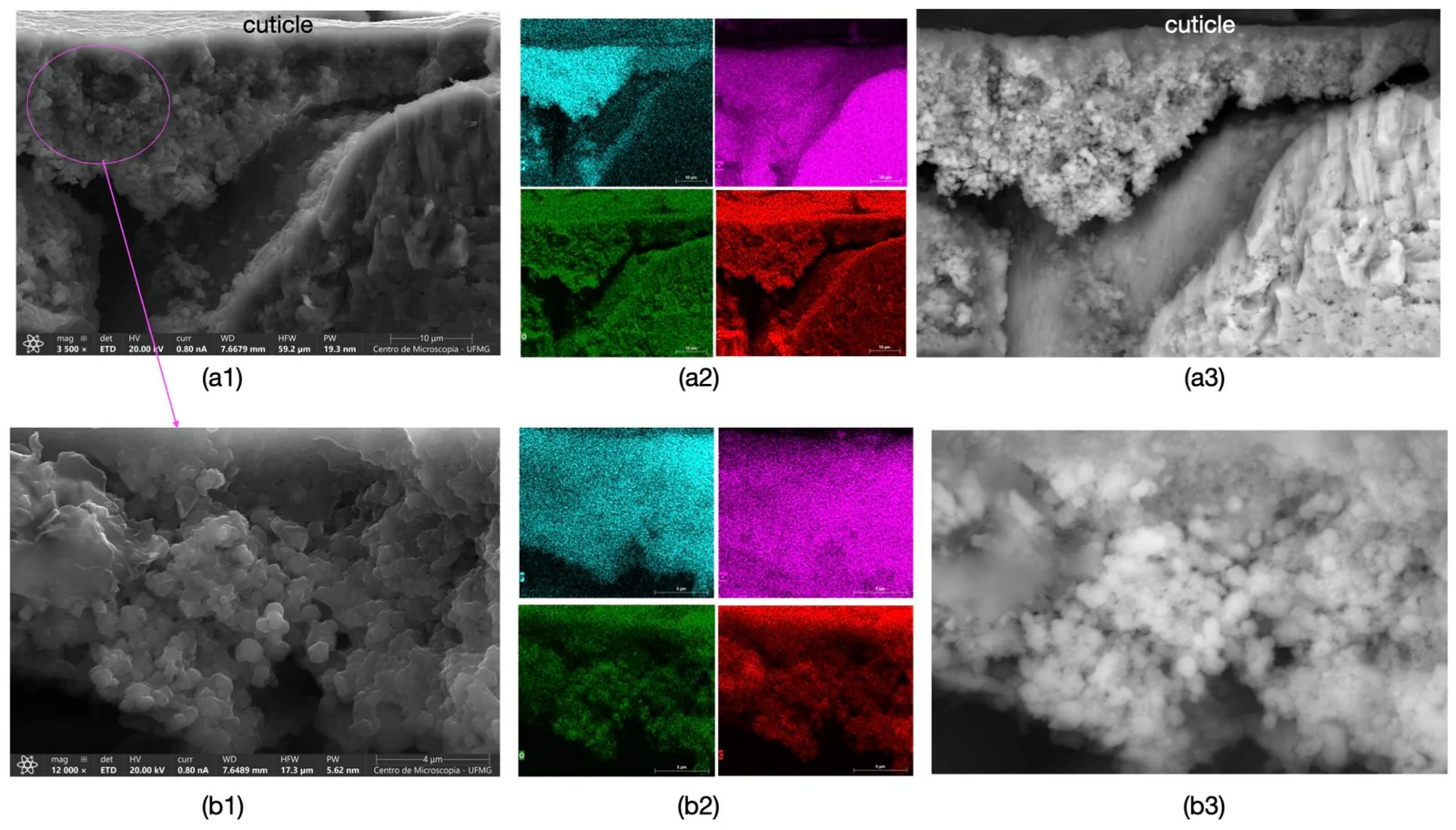
SEM images of the cuticle located exactly above a channel/pore that crosses the entire shell thickness (region of the “pore plug” [46]): (**a1**) SE detector; (**a2**) EDS map showing the distribution of phosphorus (P, light blue), calcium (Ca, magenta), oxygen (O, green), and carbon (C, red); (**a3**) same area as (**a1**) acquired with the backscattered electron (BSE) detector. (**b1**) shows a detail of (**a1**) (region circled in magenta). Spheres are visible in the images; (**b2**) EDS map showing the distribution of P, Ca, O, and C in the area presented in image (**b1**); (**b3**) BSE detector: the presence of spheres in the cuticle is clearly visible. The semi-quantitative (EDS) concentration of the elements P, Ca, O, and C present in these areas is presented in Table 2. In (**a1**,**a3**), the spheres are arranged as a “cluster of grapes.” This “cluster” occurs, as observed above, in the region of channels extending from the inner membrane to the cuticle (shown in the center of the whole-shell images in Figure 1).

The images in Figure 4 show the cuticle and the cuticle (C) together with the vertical layer (VL), with Figure 4a showing in detail the spheres in this layer. The spheres on the eggshell surface have dimensions close to 300 nm and would have, among other functions, protection against solar radiation and infectious agents [29,48]. Also in Figure 4a it is observed that smaller spheres are located throughout the cuticle but predominate on the lower face. The diameter of these smaller spheres was evaluated as (96 ± 52) nm and is shown in Table 3 and Figure 10. Figure 4b (BSE sensor) shows both the cuticle region and part of the VL, whose thickness was evaluated as (10 ± 2) μm. The fibrillar aspect of the VL and its interaction with the cuticle (C) nanospheres can be observed. Figure 4(c1) and Figure 4(c2) are, respectively, SE (topography) and BSE images showing both the VL and part of the palisade layer (PL). The PL presents plates, pores, and different growth directions of the calcitic layer, whereas the VL presents essentially a single direction, possibly due to its thickness of only about a few micrometers.

#### 3.1.1. Hemispheres (Nanodroplets Before Solidification) in the Vertical (VL) and Palisade (PL) Layers

Figure 5a,b are topographic (SE), whereas Figure 5(b1) was acquired with the BSE sensor. A white circle encloses some nano-hemispheres (and which had previously existed as nanodroplets before solidification) adhered to the plates of the vertical layer (VL). The discovery of these morphologies occurred when we were investigating palisade pores. These nanodroplets appear to indicate the shell-thickening route, which occurs by deposition and coalescence of nanodroplets (visible inside and outside the white circles in Figure 5a,b) in both the VL and the palisade PL (Figure 5c,d, topography). The diameters of these nanodroplets or nano-hemispheres (Table 3 and Figure 10) were evaluated as (88 ± 29) nm in the VL and (57 ± 12) nm in the PL. After coalescence, diameters are larger. Figure 5(b1,d1) were acquired with the BSE sensor and correspond to the same scene (yellow bidirectional arrows), evidencing structures present in areas identical to those of Figure 5b,d. It is possible to observe, especially in Figure 5(b1), that the plates seen in Figure 5b are formed by unidimensional (fibrous) calcite structures that grow through mutual adhesion of these nano-hemispheres.

We call them nanodroplets because, in the liquid environment of the laying hen uterus, these nanospheres, upon adhering to the already solidified structure, assume a droplet-like shape attached to the substrate.

There are nano-hemispheres even in the cuticle–vertical layer (VL) interaction region. These nano-hemispheres or nanodroplets must therefore contain both calcium carbonate (ACC?) and liquid, since they are deformable and coalesced on the pre-existing surface. In the already formed palisade PL, fiber density is much higher than in the VL, as can be seen by comparing Figure 5(d1) with Figure 5(b1). In Figure 5(d1), for example, it is only possible to distinguish lower fiber density at the pore edges (upper part at the center of Figure 5(d1)).

**Figure 4:**
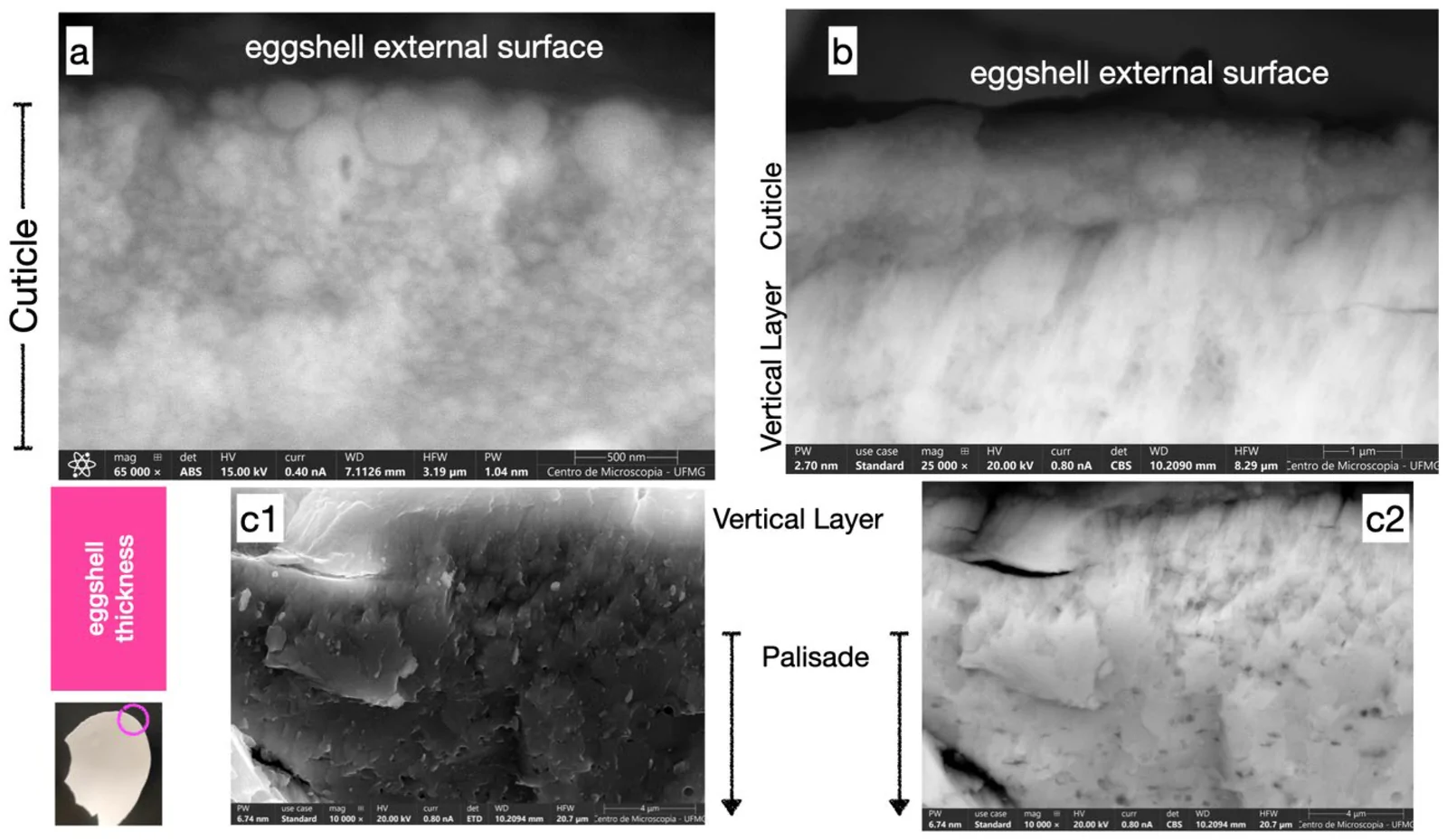
SEM images of the shell thickness: (**a**) Cuticle surface (BSE) showing larger spheres (~300 nm) on the surface. The diameters of the smaller spheres, nanospheres, were evaluated as (65 ± 24) nm. (**b**) Cuticle (thickness = 1.9 ± 0.4 μm) and part of the vertical layer (VL). The fibrillar aspect of the vertical layer is visible in the BSE image. (**c1**) Vertical layer VL (thickness = 10 ± 2 μm) and part of the palisade layer (PL). (**c2**) Same image, BSE detector. The palisade (lower part of the image) appears compact both in the surface (SE) image and in the BSE image.

**Figure 5:**
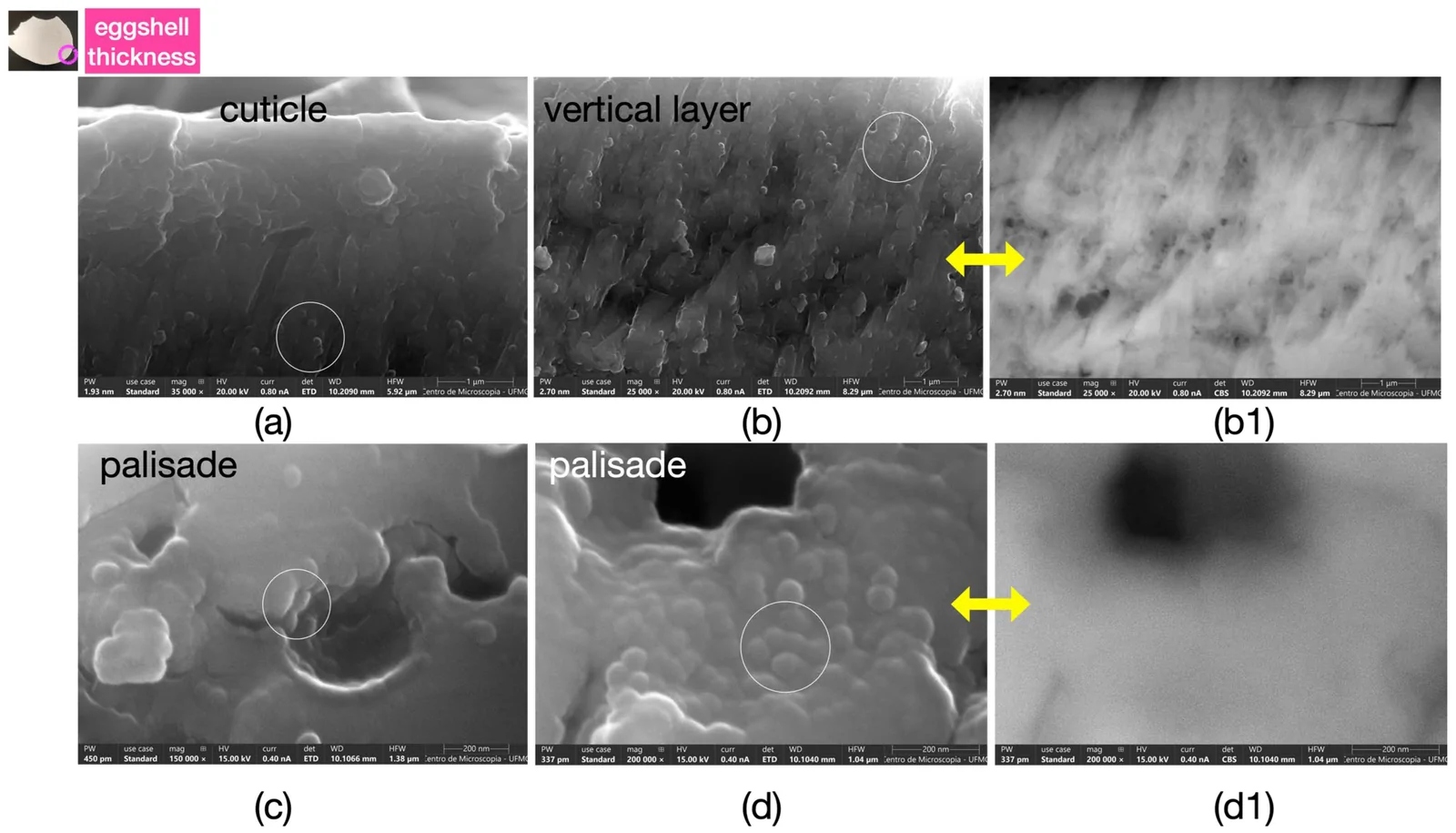
SEM images showing details of eggshell thickening growth occurring through deposition and coalescence of nano-hemispheres (marked with white circles), located both in the vertical layer (**a**,**b**) and in the palisade layer (**c**,**d**). Nano-hemispheres have diameters approximately between 50 and 90 nm. After coalescence, the diameters are larger. Images (**a**–**d**) are topographic images acquired using the SE (secondary electrons) technique. Images (**b1**,**d1**) were acquired using the BSE (backscattered electrons) technique, which indicates compositional differences, and correspond to areas identical to those shown in images b and d. It is possible to observe, especially in (**b1**), that the plates seen in image b contain unidimensional entities (fibers) of calcite, with fiber diameters on the order of a few nanometers. The nano-hemispheres must therefore contain both calcium carbonate (ACC?) and organic material (proteins and polysaccharides?). In the palisade PL, the density of calcium carbonate appears much higher than in the vertical layer, as can be concluded by comparing figure (**d1**) with (**b1**).

Figure 6a,b show the palisade layer (PL). The total thickness of the PL was evaluated as (274 ± 5) μm (Table 1) and represents nearly 80% of total shell thickness. In the palisade PL, lamellae grow over one another and in different directions. For example, in image 6a the darker gray region (lower left) grows almost perpendicular to the growth of the lamellae present in the remainder of the image. A detail of this region is shown in Figure 6b. The nanometric lamellae thickness, in the samples investigated in this work, was evaluated (see Table 3) as (30 ± 12) nm. The high standard deviation of these measurements results from the difficulty in individualizing lamella thickness. Pink asterisks in Figure 6b highlight how thin (and nearly transparent) these nanometric structures are. Even in an SE topography image, which captures electrons from depths < 10 nm, it is possible to visualize details below the lamella. In Figure 6c (BSE image), this lamella transparency due to their minute thickness is more evident because of the greater depth captured by the BSE sensor. It was not possible to evaluate lamella width, and they appear to vary in density (or composition) along their width.

Figure 7 presents images of the palisade PL region showing growth of layers in different directions and senses (Figure 7a). Black points in Figure 6a correspond to pores already seen in Figure 5a–c. Figure 7b evidences a large number of circular pores—closed or communicating with adjacent pores—present throughout the region where calcite fibrous growth occurs. From the images, it is not possible to observe any preferential porosity orientation. Comparing the pores inside the red circle in Figure 7c (SE, topography) with the same pores (red circle in Figure 7d) obtained with the BSE sensor, it is observed that the pores cross dozens of lamellae, have nearly spherical morphology, and represent—together with the channels shown in Figure 1—a considerable fraction of the shell volume [40,49,50].

Figure 8a shows a palisade PL region containing several pores. The white numbering 1, 2, and 3 in Figure 8a indicates regions shown in detail in Figure 8b–d (SE, topography). Figure 8(b′–d′) correspond to the same areas but acquired with the BSE sensor. The nearly spherical pores form concomitantly with the growth process of superposed lamellae. In Figure 8(c′) (BSE), lower right corner, it is possible to observe the presence of a pore in the lower left corner of the image that was later covered by superposed lamellae (Figure 8c). In Figure 8(d′), a void located at mid-height of the right border is not visible in Figure 8d because it was covered by superposed lamellae. Pore edges in Figure 8b–d are filled with nano-hemispheres or nanodroplets that coalesced to form a new calcite lamella. In the PL, the individual diameters of these hemispheres were evaluated as (57 ± 12) nm (see Table 3 and the plot in Figure 10).

We named them nanodroplets because they were viscous and deformable suspensions during the process of calcite lamella growth, which proceeds by additive growth through nanodroplet coalescence in the palisade layer PL and in the VL. In Figure 8b, upper left corner (just below the letter b), lamella growth occurs by coalescence of these nano-hemispheres. In Figure 8c, in the lower part of the image, nanodroplets are coalescing and forming superposed layers. In Figure 8d, this lamella growth by coalescence of nanodroplets is also observed, with a lamella growing over incomplete underlying lamellae (located at mid-height of the right border of the image).

**Figure 6:**
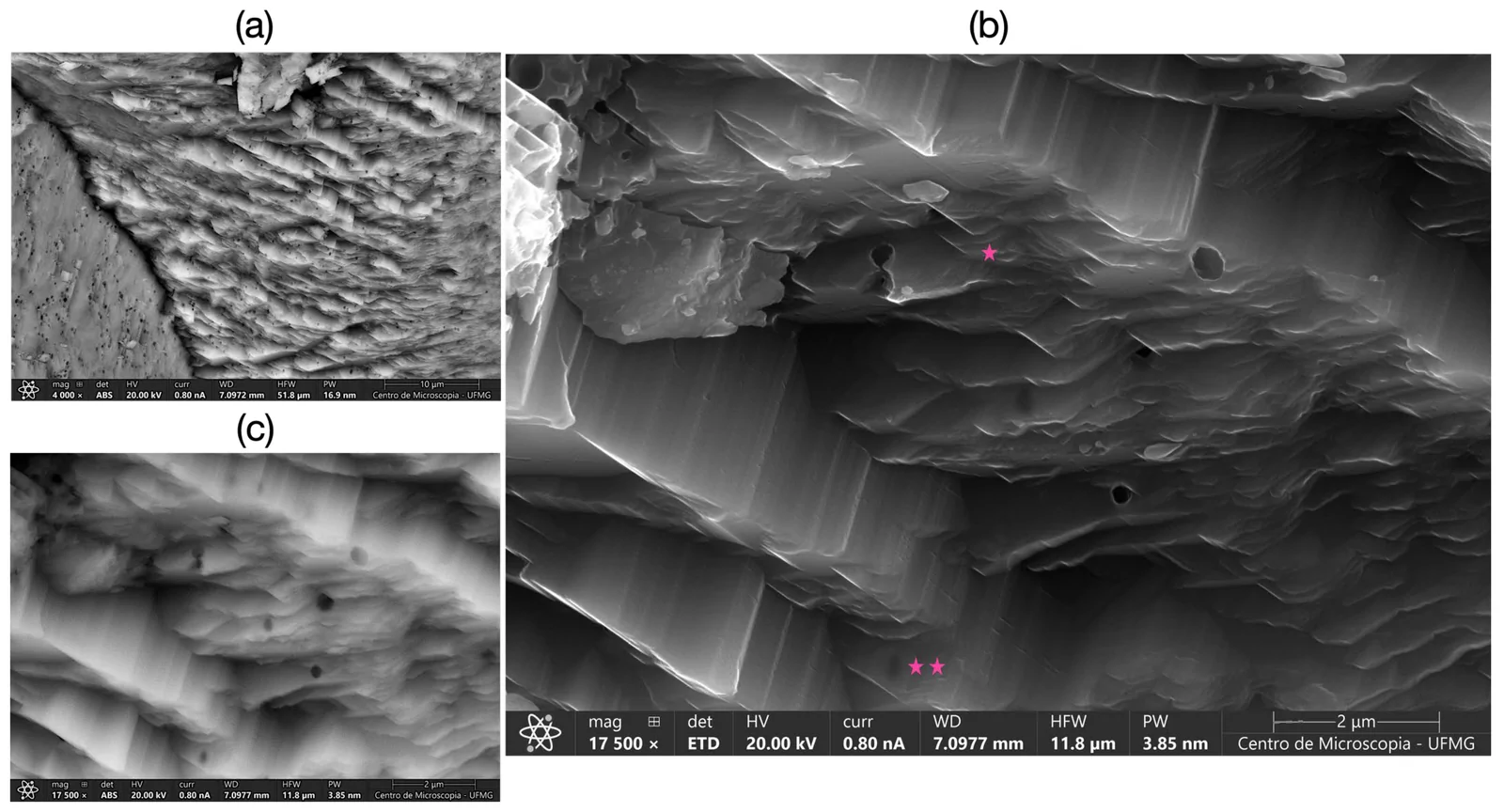
(**a**) Fracture image of the eggshell in the palisade layer (PL) region showing nanometric-thickness lamellae evaluated as 30 ± 12 nm. A large number (possibly several hundreds) of individualized and superposed lamellae are observed on the right side of the image. Note the enormous number of pores within the lamellae; (**b**) Detail of the superposed lamellae in the PL. Because they are so thin (pink star), some lamellae are transparent, revealing underlying pores (double stars) even in SE mode, which captures the electron beam at depths of 1–5 nm within the sample. The unidimensional and anisotropic growth, in fibers, as shown in Figure 4b and Figure 5(b1), possibly adhered to each other by surface interactions, water, and other substances, forms the lamellae observed in the images. Fiber growth appears to be consistent both with the speed of shell biomineralization and with its mechanical resistance; (**c**) Same image as (**b**), but acquired with the BSE detector. Lamella transparency is greater due to the deeper beam penetration captured by the BSE sensor.

**Figure 7:**
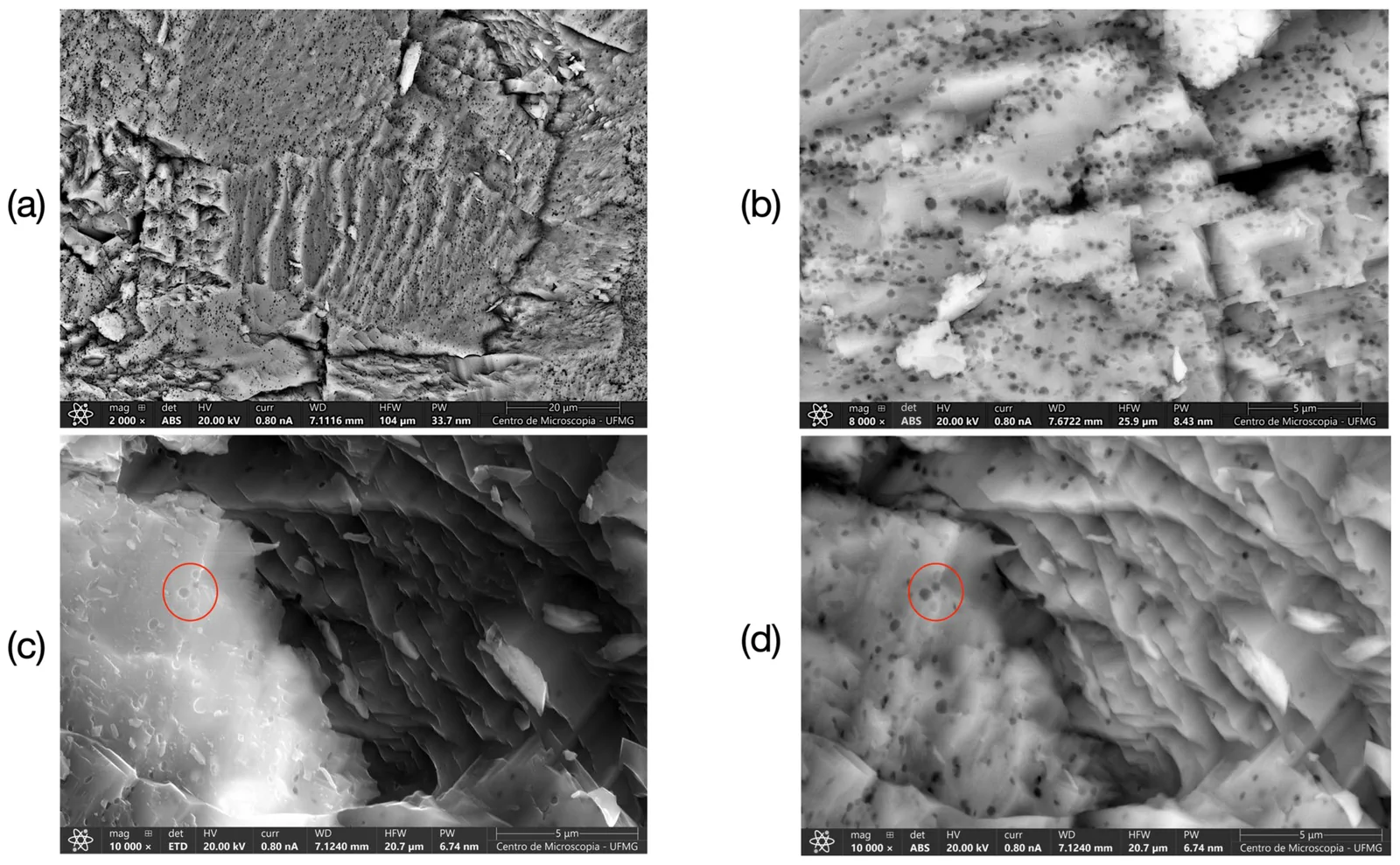
(**a**) Image, acquired using the BSE sensor, of a fractured shell area with the crystallized calcite region showing a significant number of circular pores; (**b**) High porosity of the calcite layer. An evaluation of porosity (P) presented in the two-dimensional image indicated 20% < P < 33% (Figure S8 in the Supplementary Materials); (**c**,**d**) are images of the same calcite area, with (**c**) acquired using the SE sensor, highlighting topography, and (**d**) acquired using the BSE sensor with greater depth. The pores occur in the calcite growth plane. Within the red circle (left side of the figure), at least four pores are clearly visible. Note that the pores have approximately circular shapes. Two pores appear deeper (darker), as shown in (**d**), while two are shallower, indicating that they are spheres cut (by fracture) at different heights. Pores exist where previously there was a liquid or a gas. In this case, possibly liquid, because biomineralization occurred in the uterus, in an aqueous environment. However, calcite growth was not interrupted by the pores, as evidenced by the images in Figure 5 and Figure 8 (below).

**Figure 8:**
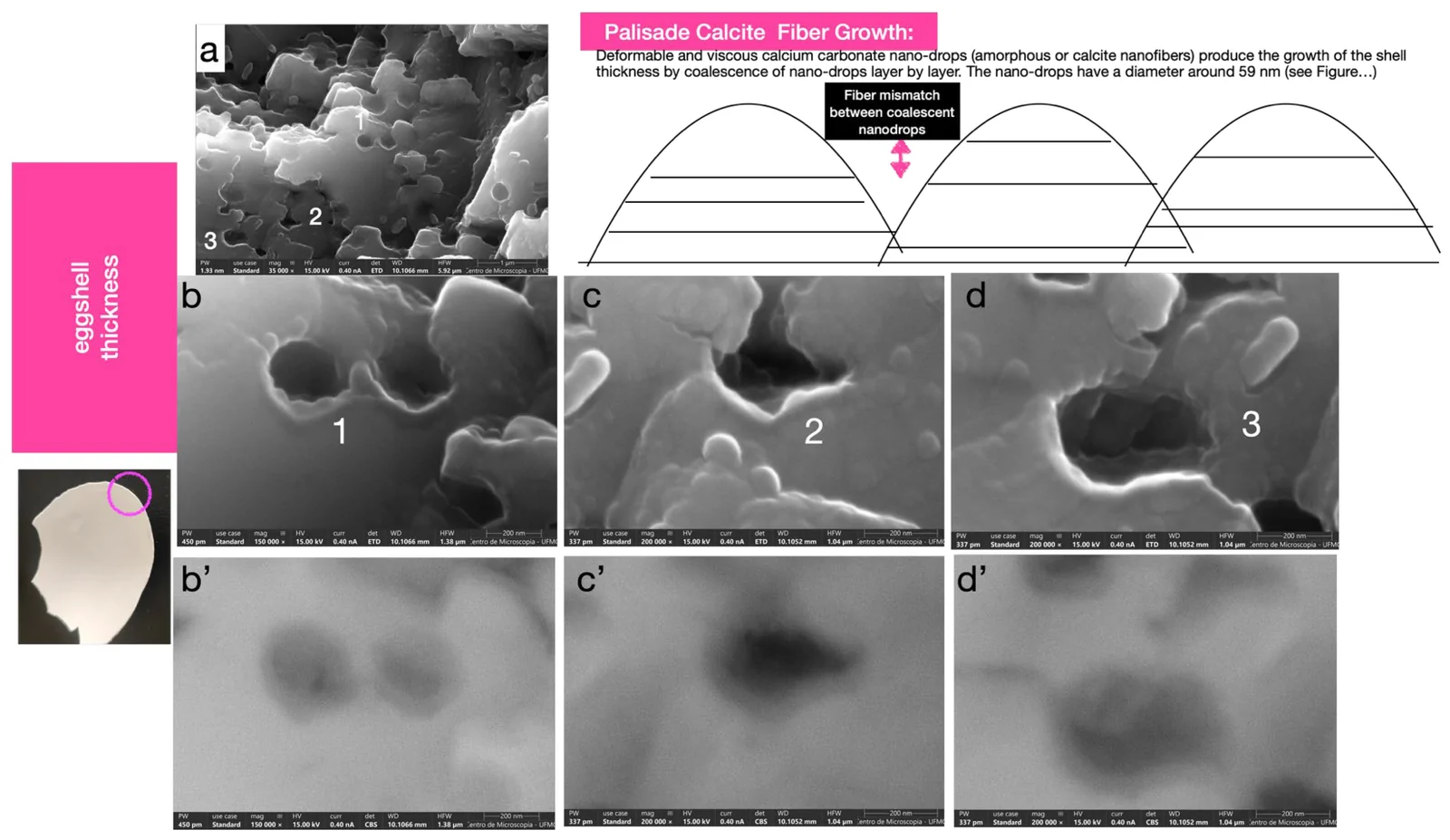
SEM images obtained across the shell thickness: (**a**) Region of the palisade layer, SE sensor, surface topography. The numbers 1, 2, and 3 represent areas shown in detail in images (**b**–**d**), also acquired using the SE sensor. Images (**b′**–**d′**) correspond, respectively, to the same areas as (**b**–**d**), but obtained using the backscattered electron (BSE) detector. By comparing the SE and BSE images, it is possible to observe that the nanodroplets are suspensions, since they are deformable and coalesce, as shown in image (**b**) at the pore edge near number 1, in image c in the cluster of nano-hemispheres near number 2, and in the coalescence of nano-hemispheres at the pore edge shown in image (**d**), near number 3. The BSE images shown in (**b′**–**d′**) indicate (especially within the pores) unidimensional growth of calcite, in fibers, as observed in the vertical layer (Figure 4b, Figure 5(b1) and Figure 6d). The presence of nano-hemispheres after biomineralization was completed indicates that there was not enough time for them to fully coalesce into a nanolayer of calcite. It may also indicate that the limiting factor is misalignment between fibers existing within each nano-hemisphere during coalescence, as suggested in the drawing (upper right part of the figure) and evidenced by the micrometric length of calcite fiber bundles shown in Figure 4b and Figure 5(b1), as well as by the different directions of calcite layers in the palisade shown in Figure 7.

When in the liquid state, did these nanospheres contain already formed fibers and liquid material (water + organic substances)? The pore edges in Figure 8(b′–d′) indicate that the interior of these hemispheres is filled with fibers whose diameters could not be individualized by SEM. However, considering the hemisphere diameter (57 ± 12) nm and the “clouds” of fibers formed at the edges in Figure 8(b′–d′), it is inferred that individual fiber diameters may approach the value of the calcite unit cell c-axis (c~1.7 nm) [51].

The content of the hemispheres shown in Figure 5 and Figure 8 likely consists of fibers, organic phase, and water. When did fiber nucleation and growth occur? Did they occur when these nanoparticles adhered to the pre-existing lamellae? Once adhered to the surface, the surface energy may be sufficient to promote heterogeneous nucleation [52] and fiber growth. However, the simultaneous coalescence of many droplets should result in fiber misalignment, as suggested by the schematic shown at the top of Figure 8.

The biomineralization rate of laying hens seems to occur more rapidly during calcite crystallization and palisade (PL) growth. This stage until laying is about 8–10 h [53] to form a layer corresponding to about 80% of eggshell thickness. Another ~8 h were spent forming the mammillary layer, whose thickness represents around 10% of eggshell thickness.

#### 3.1.2. Mammillae and Inner Membrane

The images in Figure 9 show the mammillary region using SE and BSE sensors. They correspond to exactly the same areas. The difference is that the BSE sensor captured the presence of a film enveloping the basal plate of the mammillae (Figure 9(a1)). In Figure 9b (SE, topography), we observe the growth of superposed CaCO_3_ lamellae (see the semi-quantitative composition of the area by EDS in Figure 9c) starting on the inner face of the membrane that surrounds the egg white. The same image acquired with the BSE sensor (Figure 9(b1)) shows that particles grow within the organic phase. A blue rectangle marks the same area in both images b and b1. Within the rectangle, the lamellae present on the surface of Figure 9b arise from particles that nucleated on the membrane surface. Sulfur (S) and calcium (Ca) EDS maps are shown in Figure 9(c1,c2). Sulfated organic compounds [54,55] coat the collagen membrane.

Regarding the site where calcite biomineralization begins, Figure 9(b1) and the calcium map (EDS) in Figure 9(c2) confirm what several previous and current studies have indicated: calcite crystallization occurs even within the inner membrane [8,17,56]. There, in the inner membrane, the unidimensional (fibrous) morphology of calcite crystals can also be visualized. We did not locate, in the mammillary region, the cubic calcite morphology reported by [57].

#### 3.1.3. Relationship Between Diameters of Cuticle Nanospheres and VL/PL Hemispheres

Figure 10 shows the box plot of the data in Table 3. Different lowercase letters above the boxes indicate statistically significant differences among layers according to the Kruskal–Wallis test (H = 127.08; *p* = 1.64 × 10^−26^) followed by Dunn’s post-test with Bonferroni correction (*p* < 0.05). Cuticle nanospheres and hemispheres from the vertical layer (VL) do not differ statistically. Mean diameters of cuticle spheres and VL and PL hemispheres decrease from the cuticle toward the lamella thickness. Spheres forming the pore plug (Figure 3) are larger than the other forms, possibly due to the presence of organic material increasing their diameter.

**Figure 9:**
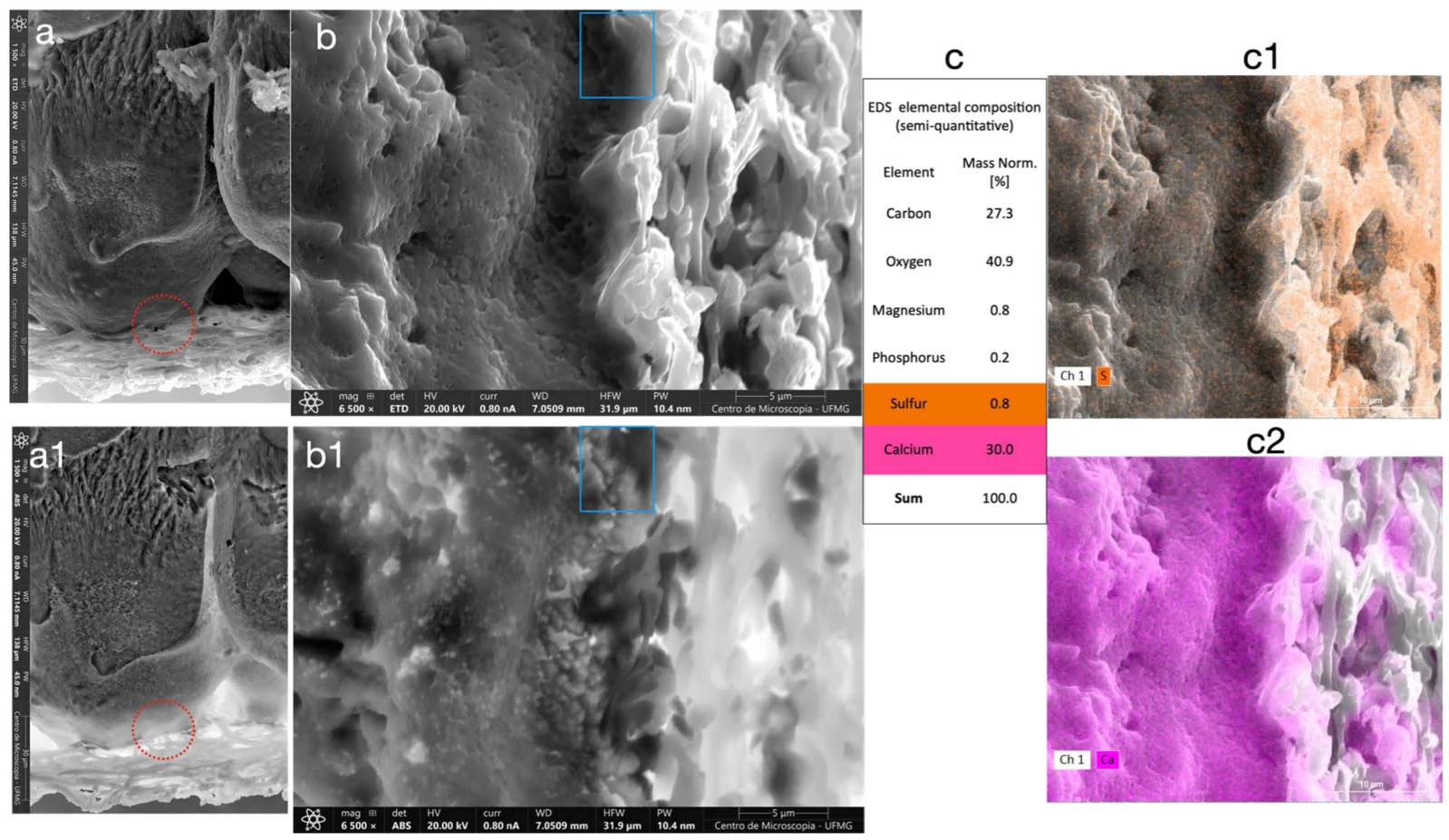
Sequence of SEM images of the same area at the base of the eggshell using SE (**a**,**b**) and BSE (**a1**,**b1**) detectors. Comparing image (**a**) (membrane, basal plate, and mammillae) with (**a1**) (BSE), it is possible to observe the existence of a layer surrounding the mammillae. This film shown in a1 is composed of lighter chemical elements (lighter elements appearing in light color), possibly an organic layer, which seems to shape the mammillae and separate them, creating space for the vertical channels/pores that cross the shell. A red dotted circle indicates the area presented in (**a**,**a1**). In the topographic image (**b**), layers growing from the membrane (left side of image (**b**)) can be observed, whereas in image (**b1**) particles are visible within the film. A blue rectangle shows that the lamellae present on the surface originate from particles that nucleated on the membrane. The table of elements (**c**) present in this area (EDS) shows the presence of sulfur (S)-containing compounds (**c1**) coating the membrane and the nucleation of CaCO_3_ (**c2**) growing over the entire outer area of the inner eggshell membrane.

**Figure 10:**
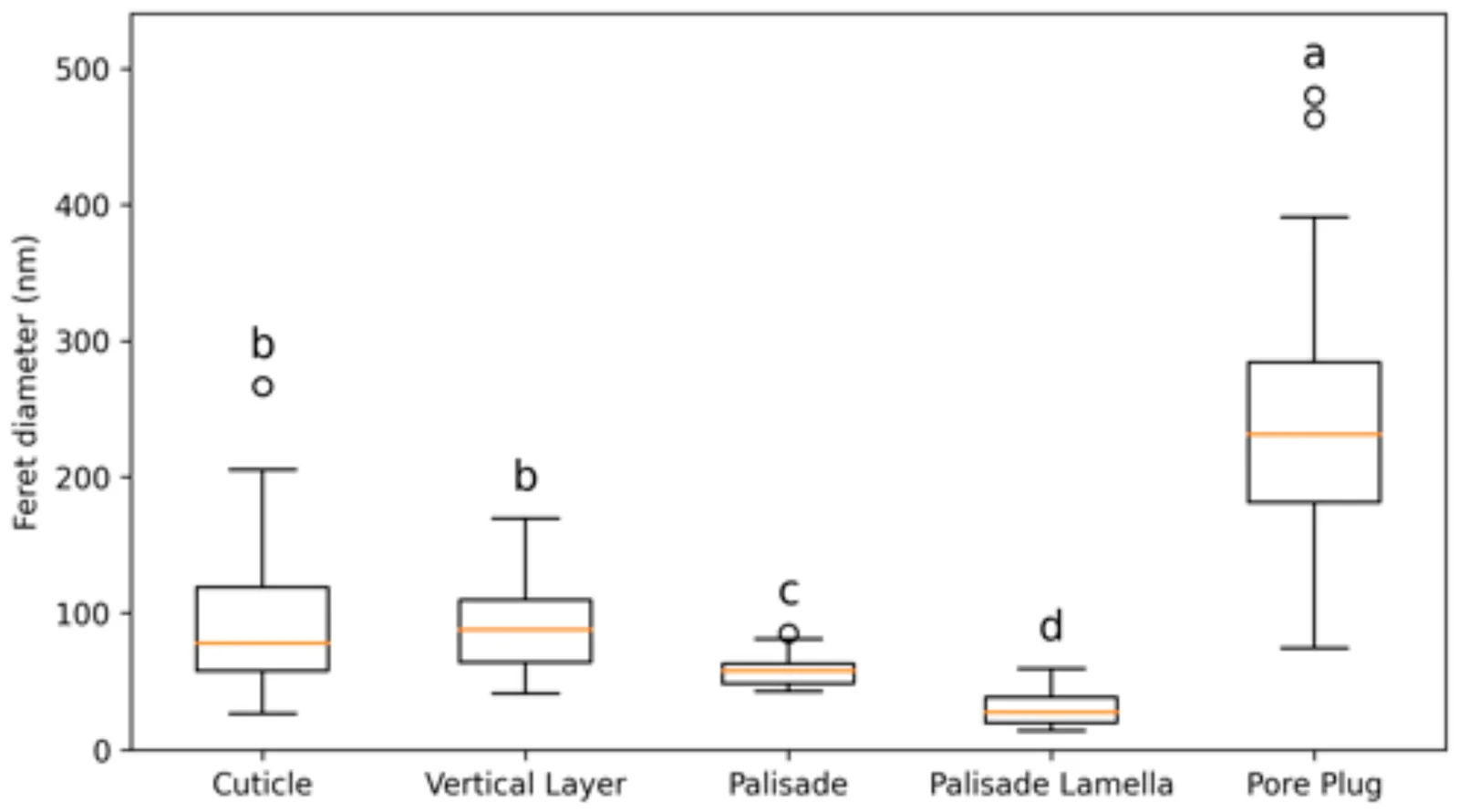
Box plot of the Feret diameter of spherical and hemispherical structures obtained from SEM images (SE mode, topography) in the cuticle (spherical), vertical layer (VL) (hemispherical), and palisade (PL) (hemispherical). Pore plug refers to the diameter of spheres present within the pore plug, and palisade lamella refers to the thickness of the individual lamellae that constitute the palisade layer. Different lowercase letters above the boxes denote statistically significant differences among layers according to the Kruskal–Wallis test followed by Dunn’s post hoc test with Bonferroni correction (*p* < 0.05).

### 3.2. Raman Spectroscopy

The Raman spectra (Witec spectrometer) focused on the cuticle region are presented in Figure 11, Figure 12 and Figure 13. Beginning with Figure 11a, which shows an image of a cross section of the shell embedded in resin, where the cuticle region is circled in red. Subsequently, Figure 11b presents the analyzed points. The spectrograms of the four points marked in Figure 11b (1–red, 2–dark blue, 3–green, and 4–light blue) are shown in Figure 11c.

In the spectrum from point 3 (green) to point 1 (red), there is an increase in the intensity of the 959 cm^−1^ peak, the most intense peak of carbonated hydroxyapatite (CHAp). The other CHAp peaks are also present, especially the 1085 cm^−1^ peak, indicative that the CHAp is B-type, that is, the apatite is carbonated by substitution of the PO_4_^3−^ group by CO_3_^2−^. CHAp-B is present in vertebrate bones.

To the left of these three points is point 4 (light blue spectrum). The main peaks marked (155, 281, 712, 739, and 1085 cm^−1^, the most intense) correspond to calcite. From the position of the points and spectra, a gradual transition from calcite (Vertical Layer, VL) to CHAp-B is observed. The spectra of points 3 (green) and 2 (dark blue) even indicate the presence of peaks at 155 and 281 cm^−1^, which decrease in intensity as the main CHAp-B peaks (959 and 1085 cm^−1^) increase. In the spectrum of point 1 (red), these peaks disappear, and only CHAp-B peaks are observed.

As already documented in the SEM images—especially in Figure 2(b3) and Figure 4b (showing the cuticle–vertical layer VL region)—and indicated by the micro-Raman spectra in Figure 11c, the CHAp-B layer—does not abruptly terminate the calcite VL. Rather, an interaction between the cuticle and the VL is observed, indicating a continuous transition from CHAp-B to calcite.

In another experiment (Figure 12), performed on a different Raman instrument (Horiba) to identify the inorganic compounds in the cuticle, two distinct preparations were produced. The spectrum in Figure 12a, obtained at the red cross, shows the shell surface with the cuticle (brown) sanded until the calcite layer (white) appeared. Figure 12b shows the Raman spectrum of the cross section of the eggshell (red cross in Figure 12b) sample. The spectrum in Figure 12b corresponds to carbonated hydroxyapatite CHAp-B.

Figure 13 presents Raman spectra (Horiba) of the external (black) and internal (red) surfaces of a chicken femur fragment (see Materials and Methods). The spectra are identical to each other and indicate CHAp-B as the biomineralized phase. The spectrum is identical to that observed in the eggshell cuticle (light blue line).

### 3.3. Infrared Spectroscopy (FTIR-ATR)

The spectra obtained using FTIR-ATR coupled to an optical microscope (micro-FTIR) in Figure 14a, recorded in the 700–1700 cm^−1^ range, compare the cuticle profile (red line) with the spectrum (black line) obtained from the internal surface of a chicken femur fragment (see item Section 2.1.3 in Section 2).

The absorption band occurring in both samples (1026 cm^−1^ for femur and 1033 cm^−1^ for cuticle, marked in the spectrum) corresponds to the main hydroxyapatite band. Some studies suggest the band in the interval 1000–1100 cm^−1^ [58,59,60,61].

Figure 14b shows the spectrum obtained in the palisade layer (PL) region (black line) and that of the cuticle (red line). The PL spectrum corresponds to calcite, with the two main peaks (712 and 872 cm^−1^, marked in Figure 14b) intense and well defined [62]. The cuticle spectrum also presents these peaks (712 and 872 cm^−1^), but with lower intensity, since the main absorption band is at 1033 cm^−1^, attributed to hydroxyapatite.

Figures S4 and S5 (Supplementary Materials) show FTIR spectra of eggshell samples previously thermally treated at 950 °C, exhibiting an absorption band between 1000 and 1100 cm^−1^ centered at ~1033 cm^−1^, which coincides with the main hydroxyapatite band [61]. Calcination of calcite at 950 °C greatly reduces the intensity of the main calcium carbonate peaks (712, 872, and 1407 cm^−1^), facilitating visualization of the main hydroxyapatite absorption band. Anhydrous calcium oxide (CaO) [63], resulting from calcite calcination, presents bands only below 400 cm^−1^.

## 4. Discussion and Conclusions

The present study provides several lines of experimental evidence concerning eggshell biomineralization. Although the observations are consistent across the different techniques employed, they do not yet support a conclusive model of the entire eggshell formation process. Some limitations should therefore be considered when interpreting the results. In particular, the sampling was not designed to assess the effects of biological and husbandry-related factors, such as genetic background, hen age and diet, flock of origin, or inter-egg variability. In addition, sample preparation may introduce artifacts that do not reflect the native structure, particularly in SEM observations and local EDS analyses.

It is therefore important to distinguish direct experimental observations reported in the Results from mechanistic interpretations of biomineralization. The latter should be regarded as hypotheses supported by the present evidence but requiring further experiments and independent sampling for confirmation.

Nevertheless, our interpretation is supported by the well-established hierarchical organization of the eggshell [64]. Previous studies have described a conserved sequence of structural layers across avian species, with related hierarchical features also reported in reptilian and fossil eggs [65]. This organization is accompanied by a characteristic mineral composition, with calcite as the predominant calcium carbonate polymorph in avian eggshells and aragonite in chelonian eggshells. Studies of wild bird eggshells have likewise shown a conserved microstructural organization despite variations in the relative thickness of individual layers [66].

The observations reported here are consistent with this established structural organization. The images presented in the Results reveal the same fundamental layers previously described in avian eggshells. Of particular relevance is the preserved cuticle architecture, comprising a predominantly organic outer region and an inner region containing inorganic components. Thus, although the proposed roles of hydroxyapatite and the mechanisms of calcite growth require further experimental validation, the structures underlying these interpretations are directly documented here and are consistent with the established hierarchical organization of the avian eggshell.

A detailed analysis of the layers of the laying hen eggshell was carried out by SEM. Special attention was given to comparing topographic images (SE sensor) with images obtained using the BSE sensor, which reaches depths of tens of micrometers and differentiates, by tonal variations, the chemical composition of the scene. The semi-quantitative values of this composition are provided by EDS [67] coupled to SEM. Other spectroscopic techniques with micrometer resolution (micro-Raman and micro-FTIR) were essential to investigate the region where the cuticle and the vertical layer (VL) come into contact.

Through this approach, we obtained several observations on eggshell formation, nearly all of which are novel. We categorized these observations, beginning with those directly supported by experimental evidence. Specifically, we observed that:

1. Well-defined spheres are present in the cuticle containing phosphorus (P) compounds, with larger diameters in the upper part and smaller diameters in the lower part. Especially in these spheres of the lower layer, which interact with the VL, the P signal is higher. Viewed in detail, these spherical nanovesicles contain, within their interior, aggregates where the P compound is located. The association between extracellular vesicles (EVs), isolated from uterine fluid, and eggshell biomineralization was first proposed by Stapane et al. [47] and more recently by Gao et al. [68].

2. Cuticle–Vertical Layer (VL): No boundary was observed between the base of the cuticle and the calcite vertical layer (VL). A clear interaction was observed with the contents of the cuticle vesicles. The interaction is mechanically demonstrated by fracture that occurs in the VL but keeps the VL adhered to the cuticle intact (Figure 2). In BSE images it became evident that the VL is composed of fibers with diameters of a few nanometers (Figure 4). The fibrous morphology of calcite is well documented in biomineralization processes involving calcite [69,70,71]. To the best of our knowledge, however, this morphology (fibers and unidirectional growth) has not previously been described in the eggshell of the laying hen. Furthermore, the absence of a distinct boundary between the cuticle and the vertical layer (VL) indicates that the role of phosphate is not to terminate the eggshell biomineralization process.

3. Vertical Layer (VL): SEM secondary electron (SE) images reveal the VL as a lamellar structure (Figure 5a,b). In contrast, the BSE image shows that these lamellae are composed of fibers that grow through interaction with the cuticle. Bubble-shaped pores are present in the VL and exhibit the same morphology as those observed in the palisade layer (PL). The occurrence of spherical pores has attracted considerable attention from researchers [37,38,39,40,41,42]; however, a satisfactory explanation for their morphology, abundance, and spatial distribution is still lacking.

4. Palisade Layer (PL): accounts for approximately 80% of the shell thickness in the samples analyzed, is also composed of nanometric-thickness lamellae. In the palisade, the calcite lamella is the smallest unit discernible by SEM in both SE (topography) and BSE images (Figure 6). The lamellae are not morphologically homogeneous. There are lighter regions, with higher concentration of calcite fibers. As already reported in the literature, we observed high concentration of “bubble-type” pores (and of similar diameter) throughout the palisade. These “bubble-type” pores may represent more than 20% of the shell volume.

5. Calcite biomineralization in the mammilla/membrane region of the inner eggshell (Figure 9(b,b1)) appears to occur within a viscous phase, which is the uterine environment. Calcite crystals appear embedded in the organic matrix. In Figure 9(c2) it is possible to observe that calcite occurs even within the inner eggshell membrane.

6. Raman spectra in the cuticle indicated the presence of B-type carbonated hydroxyapatite (CHAp-B), the same phase present in bones. Spectra obtained at micrometric points placed along a line perpendicular to the cuticle–vertical layer (VL) interface indicate continuity, with CHAp-B peaks decreasing in intensity as the points approach the vertical layer (Figure 11), until CHAp-B reduces to a minimum to give place to calcite (Figure 11c).

To confirm the presence of this type of hydroxyapatite, another experiment, using another micro-Raman spectrometer (Figure 12), indicated the CHAp-B profile in a micrometric area of the cuticle.

7. Raman spectra of CHAp-B, present on the internal (including medullary bone) and external surfaces of chicken femur, coincide with the spectrum of CHAp-B present in the eggshell cuticle (Figure 13).

With the appropriate caution that additional experiments are still required, it is reasonable to ask whether these nanodroplet-like structures could be artifacts introduced during eggshell cleaning and drying. There is no direct evidence supporting this possibility, as the samples were only rinsed to remove adhering particles and subsequently dried to eliminate residual moisture, procedures that would not be expected to generate droplet-like structures. In addition, the samples were subjected to vacuum during surface coating for SEM analysis.

More importantly, the interpretation that the nanodroplet-like structures are not preparation artifacts is supported by the observation of nanohemispheres attached to the lamellae and, particularly, along pore edges. When examined by BSE imaging, some of these structures appear to conform to the pore edges and reveal an internal fibrous morphology. It is difficult to attribute structures displaying this level of morphological organization solely to rinsing with water followed by drying. Furthermore, thermal analysis (Figure S2, Supplementary Materials) showed that substantial mass loss from the eggshells occurred only above 250 °C, while XRD (Figure S1, Supplementary Materials) indicated calcite as the only crystalline phase detected up to 750 °C. It is therefore reasonable to infer that drying at 60 °C is unlikely to substantially alter either the calcitic structure of the eggshell or the hydroxyapatite identified in the cuticle.

Taken together, these observations suggest that the nanodroplet-like morphology was exposed upon fracture of the eggshell rather than generated during sample preparation. Accordingly, while this interpretation remains hypothetical and requires further experimental validation, we propose that:

A—Figure 5a,b suggest that lamellar growth may occur through the adhesion and coalescence of nanospheres (or nanodroplets). In the BSE images (Figure 5(d1) and Figure 8(b′–d′)), fibrous structures consistent with calcite fibers can be discerned along the pore boundaries.

The schematic in the upper right corner of Figure 8 illustrates a proposed mechanism for the growth of the Palisade Layer (PL) and Vertical Layer (VL), involving the adhesion and coalescence of nanodroplets. These nanodroplets may either contain preformed calcite fibers or provide sites for their formation by heterogeneous nucleation upon contact with the existing mineral surface. If the fibers are already formed within the nanodroplets, their length would likely be limited, since long preformed fibers could hinder alignment during nanodroplet coalescence.

B—Based on Feret diameter measurements, the cuticle nanospheres and the nanohemispheres observed in the Vertical Layer (VL) show comparable size distributions (Figure 10 and Table 3). The nanohemispheres in the Palisade Layer (PL) are smaller, while the calcite lamellae measured in the PL are thinner still. We hypothesize that these size differences may reflect progressive changes during mineralization, potentially involving a higher degree of hydration of the cuticle nanodroplets, followed by dehydration and contraction as the structures evolve toward calcite lamellae. On this basis, we propose that the phosphate-containing nanospheres of the cuticle and the nanohemispheres observed in the VL and PL may represent related stages of the biomineralization process. The VL and PL nanohemispheres may therefore act as building units contributing to shell growth through successive deposition and coalescence.

C—Before solidification, these nanodroplets are hypothesized to contain water, mineral components, and organic constituents that subsequently contribute to the eggshell ultrastructure. This would provide a mechanism by which the mineral and organic components of the composite are assembled concurrently during shell formation. The persistence of nanodroplet-like structures in the fully formed eggshell may represent incompletely transformed stages of this process, in which some nanohemispheres did not fully develop into lamellar structures. Such persistence could be consistent with the rapid rate of eggshell biomineralization. If this phenomenon is confirmed as a general feature of laying hens under high production demands, its possible relationship with eggshell mechanical properties warrants further investigation.

D—The model of vesicular transport of the mineral phase appears consistent with previous observations [47]. Given the presence of CHAp-B in the cuticle, it is plausible—although not directly demonstrated by the experimental data of this study—that hydroxyapatite, either amorphous or crystalline, could be transported to the site of shell formation within vesicles. Reliable detection of phosphorus by SEM-EDS requires particular attention to the experimental conditions, as illustrated by the tests presented in Figure S5 of the Supplementary Materials. Vesicular transport of hydroxyapatite could also provide a metabolically economical pathway: CHAp-B mobilized from the hen’s medullary bone could potentially reach the uterus through the bloodstream and be incorporated into the biomineralization process with limited additional mineral transformation.

E—Although the experimental evidence presented here is not sufficient to establish this mechanism conclusively, our results raise the possibility that the conversion of CHAp-B into CaCO_3_ during eggshell formation could simultaneously release phosphate, making it metabolically available to the bird. This hypothesis requires direct experimental validation.

F—During the initial stage of embryonic skeletal development, phosphorus is supplied, at least in part, by phosphate reserves present in the egg yolk [72,73]. The complex structure of the chorioallantoic membrane (CAM) [74,75,76] and its interactions with the eggshell are beyond the scope of the present study. During a subsequent stage of skeletal growth [75], the developing embryo mobilizes calcium through the CAM vascular system from the calcium reserve body (CRB) at the base of the mammillae and from mammillary calcite. The conversion of eggshell-derived calcite into hydroxyapatite is chemically feasible and has been extensively demonstrated in studies using eggshells as a calcium source for HAp synthesis [77], while studies employing in ovo injection (IOI) further illustrate the potential utilization of calcium and phosphorus sources during embryonic development [78]. In the context of the mechanism proposed here, the nanofibrous morphology of calcite could increase the mineral surface area available for dissolution and subsequent precipitation of CHAp-B. Thus, we hypothesize that this morphology may facilitate the calcite → CHAp-B transformation during mobilization of the eggshell mineral reservoir.

## Figures and Tables

**Figure 11:**
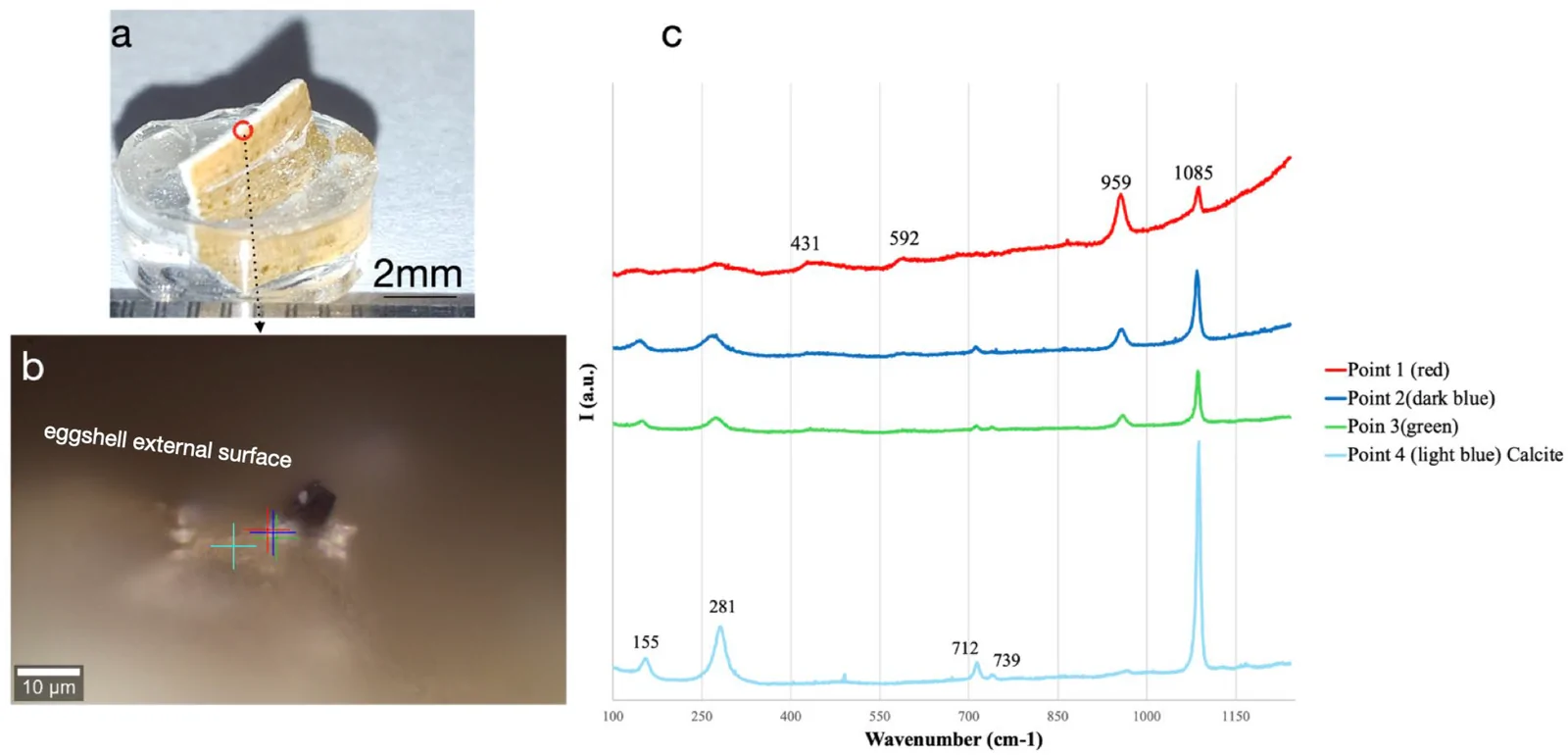
(**a**) Eggshell sample embedded in resin, with the thickness highlighted by a red circle, for investigation of the cuticle region (brown side)–vertical layer (VL) interface by micro-Raman spectroscopy; (**b**) Four points marked in the image—red (1), dark blue (2), green (3), and light blue (4)—correspond to the locations investigated in the cuticle (C)–vertical layer (VL) region, and their Raman spectra are presented in (**c**).

**Figure 12:**
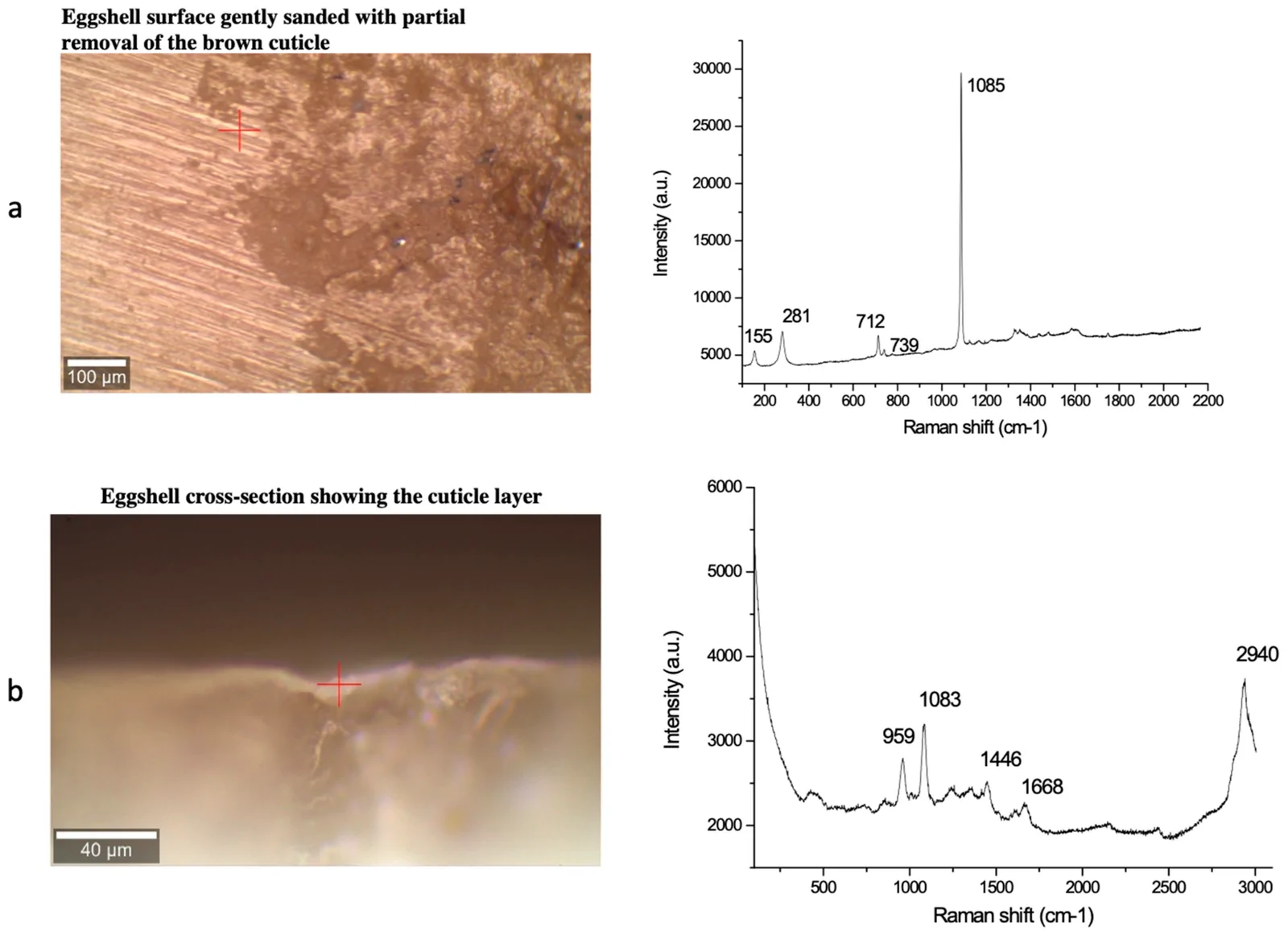
Micro-Raman analysis of two distinct sample preparations: (**a**) eggshell surface gently sanded with partial removal of the brown cuticle. The spectrum on the right was acquired at the point marked with a red cross; the spectrum corresponds to calcite. (**b**) Cross-sectional image of the eggshell in the cuticle region.

**Figure 13:**
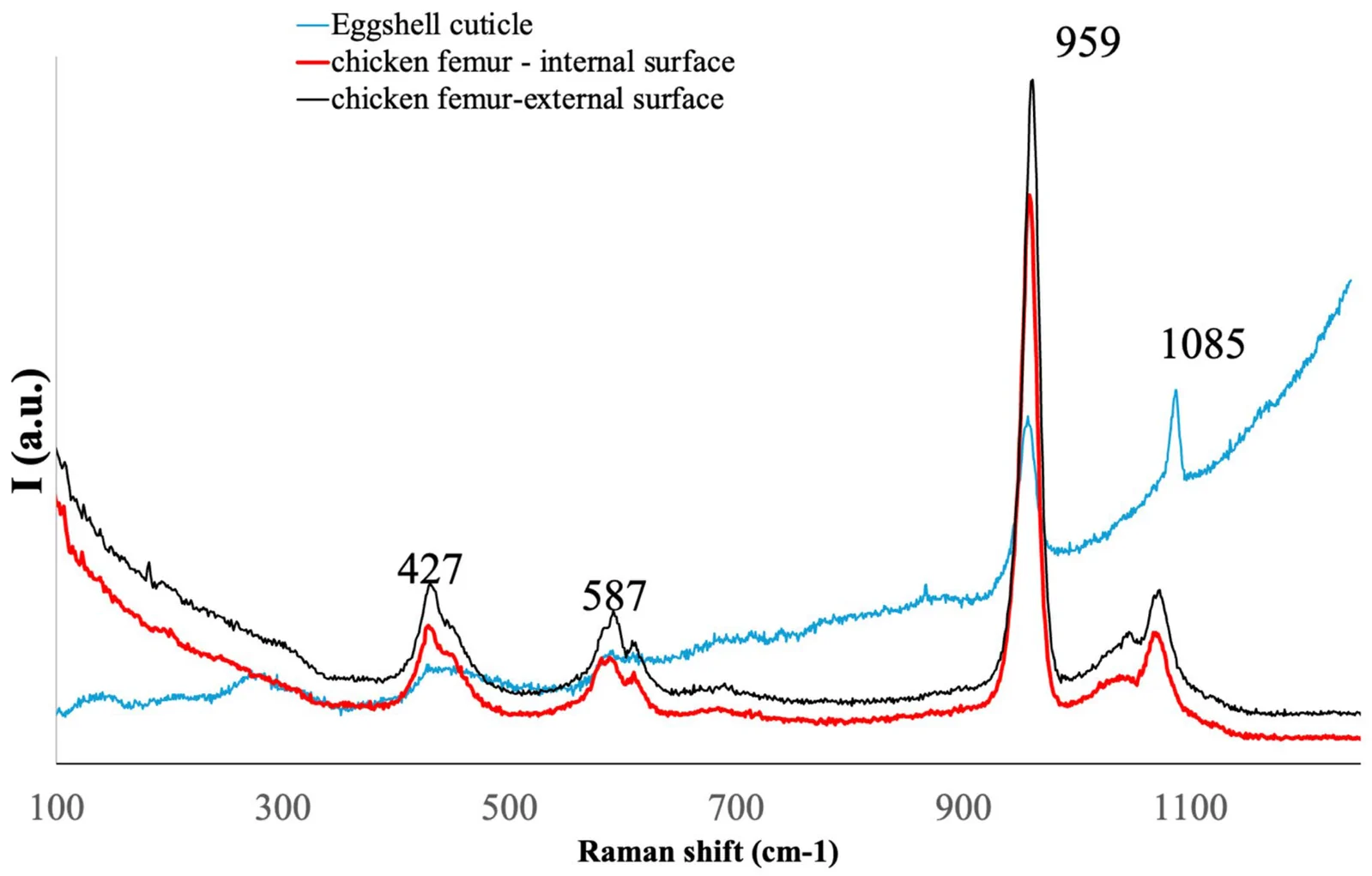
Raman spectra of the external (black line) and internal (red line) surfaces of chicken femur bone and of the eggshell cuticle (light blue line). All three exhibit the main characteristic peaks of B-type hydroxyapatite.

**Figure 14:**
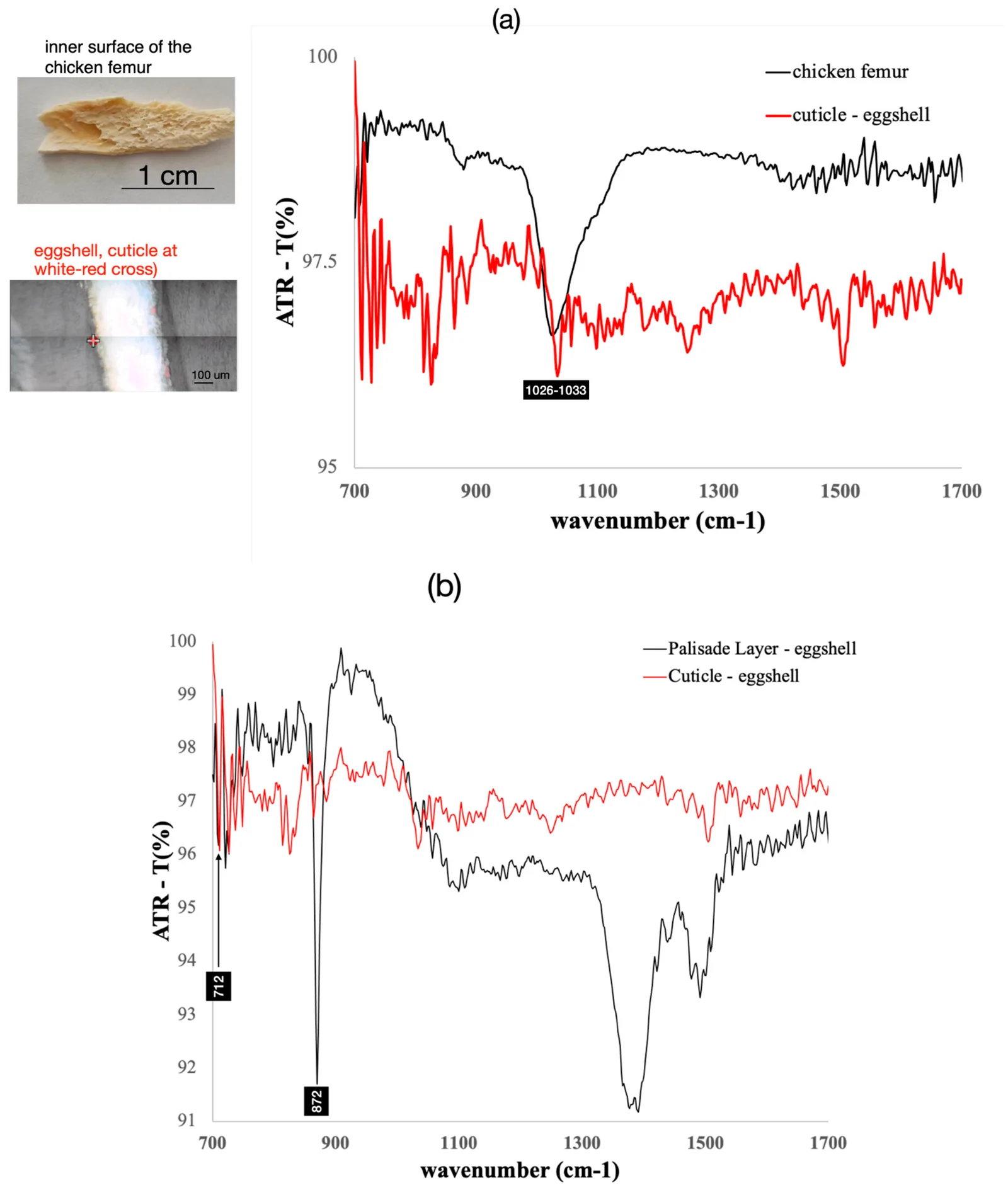
ATR-FTIR spectra (700–1700 cm^−1^, % transmittance) of: (**a**) the inner surface of the chicken femur and the eggshell cuticle (see images on the left); (**b**) the eggshell palisade layer and the cuticle. In (**a**), the main hydroxyapatite absorption band (1026–1033 cm^−1^) is observed in both the femur bone and the cuticle. In (**b**), the two characteristic calcite bands (712 and 872 cm^−1^) are clearly present in the palisade layer and are much less intense in the cuticle.

**Table 1:** Measurements of eggshell thickness (n) and its structural layers from SEM images of a single eggshell specimen using ImageJ software. The measurements presented were obtained from different SEM images of a single eggshell specimen. All measurements were performed using digital image analysis tools.

Eggshell Thickness Measurements (μm) (Feret)						
	Whole Shell	Cuticle	Vertical Layer	Palisade	Mamillary	Membrane
Thickness	353 ± 7	1.9 ± 0.4	10 ± 2	274 ± 5	22 ± 5	25 ± 4
n	10	10	12	20	33	19

**Table 2:** EDS-Elemental Mass Quantification (%).

Carbon Coating								Legend:
	#1 C at Pore Plug	#2 (C + VL)	#3 (C + VL + Part PL)	#4 (M + MA + PL)	#5 (C + VL + PL + MA + M)	#6 M	#7 M + MA	#1: Cuticle (C) #2: Cuticle(C) + Vertical Layer (VL) #3: Cuticle(C) + Vertical Layer (VL) + Part of the Palisade (PL) #4: Membrane(M) + Mamillary (MA) + Palisade (PL) #5: Cuticle(C) + Vertical Layer (VL) + Palisade (PL) + Mamillary (MA) + Membrane(M) #6: Membrane (M) #7: Membrane(M) + Mamillary (MA) NM: Not Measured
	n = 1	n = 3	n = 2	n = 2	n = 3	n = 1	n = 1	
Element		avg + std	avg	avg	avg + std			
Carbon	29.9	30.7 ± 4.3	27.8	29.7	29.2 ± 18.0	55.4	27.3	
Oxygen	32.8	27.5 ± 13.6	39.7	39.4	40.8 ± 6.0	28.1	40.9	
Magnesium	1.4	0.8 ± 0.1	0.7	0.6	0.6 ± 0.4	0.7	0.8	
Phosphorus	4.3	1.2 ± 0.1	1.2	0.1	1.4 ± 1.9	0.5	0.2	
Sulfur	NM	NM	NM	0.7	0.5 ± 0.2	1.7	0.8	
Calcium	31.5	39.9 ± 15.3	30.6	29.5	28.0 ± 14.8	13.5	30.0	
	100			100		100	100	
Gold/Palladium coating								
		#2 n = 1	#3 n = 2	#4 n = 2		#6 n = 1	#7 n = 1	
Element			avg	avg				
Carbon		6.2	8.0	10.6		45.5	17.1	
Oxygen		8.5	20.8	33.4		11.5	21.7	
Magnesium		NM	0.6	1.9		NM	NM	
Phosphorus		NM	0.5	NM		NM	NM	
Sulfur		NM	NM	NM		3.9	0.5	
Calcium		60.8	54.8	36.9		5.4	48.0	
Palladium		10.4	7.0	7.7		9.9	5.6	
Gold		14.2	8.3	9.5		10.3	5.9	

**Table 3:** Mean and standard deviation of the Feret diameter (nm) of spherical and hemispherical structures measured in the different eggshell layers. The thickness (nm) of individual lamellae constituting the palisade layer is presented in the last row of the table. The measurements were obtained from different SEM images of a single eggshell specimen. All measurements were performed using digital image analysis tools.

	Feret Diameter (nm)				
Item	Eggshell Layer	Shape	Mean	Std	Count
1	Cuticle	spheres	96	52	84
2	Vertical Layer	hemispheres	88	29	52
3	Palisade Layer	hemispheres	57	12	35
4	Pore Plug	spheres	254	110	18
		thickness (nm)			
5	Palisade Lamella	lamella	30	12	38

## Data Availability

The data supporting the results of this article will be made available by the authors on request.

## Supplementary Materials

The following supporting information can be downloaded at https://www.mdpi.com/article/10.3390/ani16193023/s1: Figure S1. X-ray diffraction (XRD) of eggshells thermally treated in air at 150 °C (blue) and 950 °C (red) for 1 h. The phase at 150 °C is calcite (CaCO_3_), whereas at 950 °C the phase is calcium oxide (CaO); Figure S2. Thermogravimetric analysis of eggshell samples previously treated at 150, 350, and 950 °C. For comparison, CaCO_3_ was also analyzed [79,80]. The tests were performed up to 600 °C with a heating rate of 10 °C/min; Figure S3. Infrared (FTIR) spectrum of eggshell samples previously treated at 150, 350, 550, 750, and 950 °C. The bands of samples treated up to 750 °C correspond to CaCO_3_ and remain present, although much less intense, in the sample treated at 950 °C. In this sample (red line), the absorption band in the 1000–1100 cm^−1^ range (centered at ~1033 cm^−1^) is not attributed to CaCO_3_ [62,81]; Figure S4. Infrared (FTIR) spectra: (i) of an eggshell sample previously treated at 950 °C (red curve) and (ii) of a bovine hydroxyapatite sample. The spectrum of the sample treated at 950 °C still exhibits bands of residual CaCO_3_; however, the band between 1000–1100 cm^−1^ partially coincides with the 900–1100 cm^−1^ band of bovine hydroxyapatite [82,83,84]; Figure S5. X-ray emission peaks obtained by EDS spectroscopy in the cuticle region from two distinct areas of a gold–palladium-coated eggshell sample (solid red and blue lines). Three dashed lines (black, purple, and green) correspond to a carbon-coated eggshell sample. The gold emission peak (2.12 keV) overlaps with the phosphorus peak (2.03 keV); Figure S6. Mammillary region that, upon detachment from the inner membrane (marked with number 2) of the eggshell, reveals the interior of the Calcium Reserve Body (CRB), marked with number 1. Spheres with diameters < 1 μm constitute this reserve. The crown, marked with number 3, indicates the beginning of the palisade layer (PL). The length (dotted line) was evaluated as (17.2 ± 2.4) μm; Figure S7. XRD spectrum of the reference hydroxyapatite material (black line) and the sample obtained from bovine bone (green dotted line) as described in item 2.1.4 of the Materials and Methods section of this work; Figure S8. Sequence (below) of eight SEM images of eggshell samples showing the continuity between the Cuticle (C) and the Vertical Layer (VL). In the arrangement shown, the images on the right were acquired using SE (a, c, e, g), whereas the images on the left were acquired using BSE (b, d, f, h).

## References

[1] Ketta M., Tůmová E. (2018). Relationship between eggshell thickness and other eggshell measurements in eggs from litter and cages. Ital. J. Anim. Sci..

[2] Yan Y.Y., Sun C.J., Lian L., Zheng J.X., Xu G.Y., Yang N. (2014). Effect of uniformity of eggshell thickness on eggshell quality in chickens. J. Poult. Sci..

[3] Sun C.J., Chen S.R., Xu G.Y., Liu X.M., Yang N. (2012). Global variation and uniformity of eggshell thickness for chicken eggs. Poult. Sci..

[4] Benavides-Reyes C., Folegatti E., Dominguez-Gasca N., Litta G., Sanchez-Rodriguez E., Rodriguez-Navarro A.B., Umar Faruk M. (2021). Research Note: Changes in eggshell quality and microstructure related to hen age during a production cycle. Poult. Sci..

[5] Alig B.N., Anderson K.E., Malheiros D.M., Harding K.L., Malheiros R.D. (2025). Assessment of the effects of stocking density on laying hens raised in colony cages: Part II—Egg production, egg quality, and welfare parameters. Poultry.

[6] Arulnathan V., Turner I., Bamber N., Ferdous J., Grassauer F., Doyon M., Pelletier N. (2024). A systematic review of potential productivity, egg quality, and animal welfare implications of extended lay cycles in commercial laying hens in Canada. Poult. Sci..

[7] Noble R., Noble D. (2023). Understanding Living Systems.

[8] Buss D.J., Reznikov N., McKee M.D. (2023). Attaching organic fibers to mineral: The case of the avian eggshell. iScience.

[9] Buss D.J., Deering J., Reznikov N., McKee M.D. (2025). Understanding the structural biology of osteomalacia through multiscale 3D X-ray and electron tomographic imaging: A review of X-linked hypophosphatemia, the Hyp mouse model, and imaging methods. JBMR Plus.

[10] Fernandez M.S., Araya M., Arias J.L. (1997). Eggshells are shaped by a precise spatio-temporal arrangement of sequentially deposited macromolecules. Matrix Biol..

[11] Leal C., Braga B., Cardoso D., Ferreira A.C., Oliveira-Bahia V., Marques J.R., Guimarães D.A. (2024). Eggshell ultrastructure and mineral composition during the osteogenesis of Kinosternon scorpioides (Testudines: Kinosternidae). Zoomorphology.

[12] Solomon S.E., Baird T. (1976). Studies on the eggshell (oviducal and oviposited) of *Chelonia mydas* L.. J. Exp. Mar. Biol. Ecol..

[13] Chang Y., Chen P.Y. (2016). Hierarchical structure and mechanical properties of snake (*Naja atra*) and turtle (*Ocadia sinensis*) eggshells. Acta Biomater..

[14] Athanasiadou D., Jiang W., Goldbaum D., Saleem A., Basu K., Pacella M.S., Böhm C.F., Chromik R.R., Hincke M.T., Rodríguez-Navarro A.B. (2018). Nanostructure, osteopontin, and mechanical properties of calcitic avian eggshell. Sci. Adv..

[15] Rodríguez-Navarro A.B., Marie P., Nys Y., Hincke M.T., Gautron J. (2015). Amorphous calcium carbonate controls avian eggshell mineralization: A new paradigm for understanding rapid eggshell calcification. J. Struct. Biol..

[16] Gautron J., Stapane L., Le Roy N., Nys Y., Rodriguez-Navarro A.B., Hincke M.T. (2021). Avian eggshell biomineralization: An update on its structure, mineralogy and protein tool kit. BMC Mol. Cell Biol..

[17] Dennis J.E., Xiao S., Agarwal M., Fink D.J., Heuer A.H., Caplan A.I. (1996). Microstructure of matrix and mineral components of eggshells from white leghorn chickens (*Gallus gallus*). J. Morphol..

[18] Smith A.H., Winget C.M., Blackard J.R. (1954). The transfer of phosphorus to the hen’s egg, under controlled environment, as traced with radiophosphorus (P32). Poult. Sci..

[19] Garcia-Mejia R.A., Sinclair-Black M., Blair L.R., Angel R., Jaramillo B., Regmi P., Neupane N., Proszkowiec-Weglarz M., Arbe X., Cavero D. (2024). Physiological changes in the regulation of calcium and phosphorus utilization that occur after the onset of egg production in commercial laying hens. Front. Physiol..

[20] Coudert E., Corrales N., Coto C., Menoyo D., Fondevila G. (2025). The role of phosphorus sources and phytase in growth performance and feed digestibility in broilers. Animals.

[21] Lotsari A., Rajasekharan A.K., Halvarsson M., Andersson M. (2018). Transformation of amorphous calcium phosphate to bone-like apatite. Nat. Commun..

[22] D’Alba L., Torres R., Waterhouse G.I.N., Eliason C., Hauber M.E., Shawkey M.D. (2017). What does the eggshell cuticle do? A functional comparison of avian eggshell cuticles. Physiol. Biochem. Zool..

[23] Rodríguez-Navarro A.B., Domínguez-Gasca N., Muñoz A., Ortega-Huertas M. (2013). Change in the chicken eggshell cuticle with hen age and egg freshness. Poult. Sci..

[24] Mahato P.L., Weatherby T., Ewell K., Jha R., Mishra B. (2024). Scanning electron microscope-based evaluation of eggshell quality. Poult. Sci..

[25] Freeman C.L., Harding J.H. (2023). The transformation of amorphous calcium carbonate to calcite and classical nucleation theory. J. Cryst. Growth.

[26] Ketta M., Tůmová E. (2016). Eggshell structure, measurements, and quality-affecting factors in laying hens: A review. Czech. J. Anim. Sci..

[27] Park J.A., Sohn S.H. (2018). The influence of hen aging on eggshell ultrastructure and shell mineral components. Korean J. Food Sci. Anim. Resour..

[28] Wedral E.M., Vadehra D.V., Baker R.C. (1974). Chemical composition of the cuticle, and the inner and outer shell membranes from eggs of Gallus gallus. Comp. Biochem. Physiol. B.

[29] Klingensmith P.M., Hester P.Y. (1983). The relationship of dietary levels of phosphorus to the production of soft-shelled and shell-less eggs. Poult. Sci..

[30] Ogasawara T., Koga O., Nishiyama H. (1975). Premature oviposition induced by intrauterine injection of phosphate solution in the laying hen. Jpn. J. Zootech. Sci..

[31] Tang C., Wei Y., Gu L., Zhang Q., Li M., Yuan G., He Y., Huang L., Liu Y., Zhang Y. (2020). Biomineral precursor formation is initiated by transporting calcium and phosphorus clusters from the endoplasmic reticulum to mitochondria. Adv. Sci..

[32] Raguin E., Weinkamer R., Schmitt C., Curcuraci L., Fratzl P. (2023). Logistics of bone mineralization in the chick embryo studied by 3D cryo FIB-SEM imaging. Adv. Sci..

[33] Sinclair-Black M., Garcia R.A., Ellestad L.E. (2023). Physiological regulation of calcium and phosphorus utilization in laying hens. Front. Physiol..

[34] Simkiss K. (1964). Phosphates as crystal poisons of calcification. Biol. Rev..

[35] Zou Z., Xie J., Macías-Sánchez E., Fu Z. (2021). Nonclassical crystallization of amorphous calcium carbonate in the presence of phosphate ions. Cryst. Growth Des..

[36] Molnár Z., Dódony I., Pósfai M. (2023). Transformation of amorphous calcium carbonate in the presence of magnesium, phosphate, and mineral surfaces. Geochim. Cosmochim. Acta.

[37] Zhou J., Wang S., Nie F., Feng L., Zhu G., Jiang L. (2011). Elaborate architecture of the hierarchical hen’s eggshell. Nano Res..

[38] Donaire M., López-Martínez N. (2009). Porosity of Late Paleocene Ornitholithus eggshells (Tremp Fm, south-central Pyrenees, Spain): Palaeoclimatic implications. Palaeogeogr. Palaeoclimatol. Palaeoecol..

[39] La Scala N., Boleli I., Ribeiro L., Freitas D., Macari M. (2000). Pore size distribution in chicken eggs as determined by mercury porosimetry. Rev. Bras. Cienc. Avic..

[40] Ahmed M.W., Alam S., Khaliduzzaman A., Emmert J.L., Kamruzzaman M. (2025). Non-destructive measurement of eggshell strength using NIR spectroscopy and explainable artificial intelligence. J. Sci. Food Agric..

[41] Riley A., Sturrock C.J., Mooney S.J., Luck M.R. (2014). Quantification of eggshell microstructure using X-ray micro computed tomography. Br. Poult. Sci..

[42] Arzate-Vázquez I., Méndez-Méndez J.V., Flores-Johnson E.A., Nicolás-Bermúdez J., Chanona-Pérez J.J., Santiago-Cortés E. (2019). Study of the porosity of calcified chicken eggshell using atomic force microscopy and image processing. Micron.

[43] Kulshreshtha G., Rodriguez-Navarro A., Sanchez-Rodriguez E., Diep T., Hincke M.T. (2018). Cuticle and pore plug properties in the table egg. Poult. Sci..

[44] Carrino D.A., Dennis J.E., Wu T.M., Arias J.L., Fernandez M.S., Rodriguez J.P., Fink D.J., Heuer A.H., Caplan A.I. (1996). The avian eggshell extracellular matrix as a model for biomineralization. Connect. Tissue Res..

[45] Hincke M.T., Nys Y., Gautron J., Mann K., Rodriguez-Navarro A.B., McKee M.D. (2012). The eggshell: Structure, composition and mineralization. Front. Biosci..

[46] Liang X., Guo S., Kuang X., Wan X., Liu L., Zhang F., Jiang G., Cong H., He H., Tan S.C. (2024). Recent advancements and perspectives on processable natural biopolymers: Cellulose, chitosan, eggshell membrane, and silk fibroin. Sci. Bull..

[47] Stapane L., Le Roy N., Ezagal J., Rodriguez-Navarro A.B., Labas V., Combes-Soia L., Hincke M.T., Gautron J. (2020). Avian eggshell formation reveals a new paradigm for vertebrate mineralization via vesicular amorphous calcium carbonate. J. Biol. Chem..

[48] Leleu S., Messens W., De Reu K., De Preter S., Herman L., Heyndrickx M., De Baerdemaeker J., Michiels C., Bain M. (2011). Effect of egg washing on the cuticle quality of brown and white table eggs. J. Food Prot..

[49] Montenegro A.T., Garcia E.A., Molino A.B., Cruvinel J.M., Ouros C.C., Alves K.S. (2019). Methods to evaluate the eggshell quality of table eggs. Braz. J. Poult. Sci..

[50] Voisey P.W., Hunt J.R. (1974). Measurement of eggshell strength. J. Texture Stud..

[51] Winchell H. (1956). The unit cells of calcite. Am. J. Sci..

[52] Abbaschian R., Abbaschian L., Reed-Hill R.E. (2010). Physical Metallurgy Principles.

[53] Fu Y., Zhou J., Schroyen M., Zhang H., Wu S., Qi G., Wang J. (2024). Decreased eggshell strength caused by impairment of uterine calcium transport coincide with higher bone minerals and quality in aged laying hens. J. Anim. Sci. Biotechnol..

[54] Fernandez M.S., Moya A., Lopez L., Arias J.L. (2001). Secretion pattern, ultrastructural localization and function of extracellular matrix molecules involved in eggshell formation. Matrix Biol..

[55] Bedini E., Corsaro M.M., Fernández-Mayoralas A., Iadonisi A., Cohen E., Merzendorfer H. (2019). Chondroitin, dermatan, heparan, and keratan sulfate: Structure and functions. Extracellular Sugar-Based Biopolymers Matrices. Biologically-Inspired Systems.

[56] Wyburn G.M., Johnston H.S., Draper M.H., Davidson M.F. (1973). The ultrastructure of the shell forming region of the oviduct and the development of the shell of Gallus domesticus. Q. J. Exp. Physiol..

[57] Heyn A.N.J. (1963). The crystalline structure of calcium carbonate in the avian eggshell: An electron microscope study. J. Ultrastruct. Res..

[58] Antonakos A., Liarokapis E., Leventouri T. (2007). Micro-Raman and FTIR studies of synthetic and natural apatites. Biomaterials.

[59] Madupalli H., Pavan B., Tecklenburg M.M.J. (2017). Carbonate substitution in the mineral component of bone: Discriminating the structural changes, simultaneously imposed by carbonate in A and B sites of apatite. J. Solid State Chem..

[60] Gupta S., Panneer Selvam S., Ramadoss R., Sundar S. (2025). Alginate-hydroxyapatite scaffolds: A comprehensive characterization study. J. Oral Biol. Craniofac. Res..

[61] Hamzah S., Yatim N.I., Alias M., Ali A., Rasit N., Abuhabib A. (2019). Extraction of hydroxyapatite from fish scales and its integration with rice husk for ammonia removal in aquaculture wastewater. Indones. J. Chem..

[62] Possenti E., Colombo C., Realini M., Song C.L., Kazarian S.G. (2021). Time-resolved ATR–FTIR spectroscopy and macro ATR–FTIR spectroscopic imaging of inorganic treatments for stone conservation. Anal. Chem..

[63] Tang Y., Xu J., Zhang J., Lu Y. (2013). Biodiesel production from vegetable oil by using modified CaO as solid basic catalysts. J. Clean. Prod..

[64] Dauphin Y., Werner D., Corado R., Perez-Huerta A. (2021). Structure and composition of the eggshell of a passerine bird, Setophaga ruticilla (Linnaeus, 1758). Microsc. Microanal..

[65] Mikhailov K.E. (1991). Classification of fossil eggshells of amniotic vertebrates. Acta Palaeontol. Pol..

[66] Damaziak K., Marzec A. (2022). Analysis of ultrastructure and microstructure of blackbird (*Turdus merula*) and song thrush (*Turdus philomelos*) eggshell by scanning electron microscopy and X-ray computed microtomography. Sci. Rep..

[67] Hajizadeh Z., Taheri-Ledari R., Rasouli Asl F., Maleki A. (2022). Identification and analytical methods. Heterogeneous Micro and Nanoscale Composites for the Catalysis of Organic Reactions.

[68] Gao L.B., Fu Y., Guo Y.T., Zhang H.J., Qi G.H., Wang J. (2025). Proteomic changes in extracellular vesicle and uterine fluid proteins correlated with eggshell quality in aged laying hens. Poult. Sci..

[69] Kniprath E. (1974). Ultrastructure and growth of the sea urchin tooth. Calcif. Tissue Res..

[70] Brear K., Currey J.D. (1976). Structure of a sea urchin tooth. J. Mater. Sci..

[71] Villagran X.S., Poch R.M. (2014). A new form of needle-fiber calcite produced by physical weathering of shells. Geoderma.

[72] Halgrain M., Bernardet N., Crepeau M., Même N., Narcy A., Hincke M., Réhault-Godbert S. (2022). Eggshell decalcification and skeletal mineralization during chicken embryonic development: Defining candidate genes in the chorioallantoic membrane. Poult. Sci..

[73] Chen L., Wang S., Feng Y., Zhang J., Du Y., Zhang J., Van Ongeval C., Ni Y., Li Y. (2021). Utilisation of chick embryo chorioallantoic membrane as a model platform for imaging-navigated biomedical research. Cells.

[74] Saran U., Gemini Piperni S., Chatterjee S. (2014). Role of angiogenesis in bone repair. Arch. Biochem. Biophys..

[75] Deickert J.W., Dieckert M.C., Creger C.R. (1989). Calcium reserve assembly: A basic structural unit of the calcium reserve system of the hen eggshell. Poult. Sci..

[76] Dhayer M., Jordao A., Dekiouk S., Cleret D., Germain N., Marchetti P. (2024). Implementing chicken chorioallantoic membrane (CAM) assays for validating biomaterials in tissue engineering: Rationale and methods. J. Biomed. Mater. Res. B Appl. Biomater..

[77] Zhang Y., Pham H.M., Tran S.D. (2024). The chicken egg: An advanced material for tissue engineering. Biomolecules.

[78] Matuszewski A., Łukasiewicz M., Niemiec J., Kamaszewski M., Jaworski S., Domino M., Jasiński T., Chwalibog A., Sawosz E. (2021). Calcium carbonate nanoparticles—Toxicity and effect of in ovo inoculation on chicken embryo development, broiler performance and bone status. Animals.

[79] Benataya K., Lakrat M., Elansari L.L., Mejdoubi E. (2020). Synthesis of B-type carbonated hydroxyapatite by a new dissolution-precipitation method. Mater. Today Proc..

[80] Ion R.M., Iancu L., Vasilievici G., Grigore M.E., Andrei R.E., Radu G.I., Grigorescu R.M., Teodorescu S., Bucurica I.A., Ion M.L. (2019). Ion-substituted carbonated hydroxyapatite coatings for model stone samples. Coatings.

[81] Campbell S., Gao B., Poduska K.M. (2020). Mid- and far-infrared spectral links for calcium carbonate polymorphs. Proceedings of the 2020 45th International Conference on Infrared, Millimeter, and Terahertz Waves (IRMMW-THz).

[82] Belaidouni A., Dali-Youcef Z., Attar T., Bekheira S., Belbal R. (2022). Removal of phosphates in aqueous solution by adsorption on calcium oxide. Fr.-Ukr. J. Chem..

[83] Tangboriboon N., Phudkrachang P., Mulsow L.O., Kunchornsup W., Sirivat A. (2013). Removal of water extractable proteins from concentrated natural rubber latex by eggshells. J. Elastomers Plast..

[84] Mohadi R., Sueb A., Anggraini K., Lesbani A. (2018). Calcium oxide catalyst based on quail eggshell for biodiesel synthesis from waste palm oil. J. Pure Appl. Chem. Res..

